# The effects of pre-task music on exercise performance and associated psycho-physiological responses: a systematic review with multilevel meta-analysis of controlled studies

**DOI:** 10.3389/fpsyg.2023.1293783

**Published:** 2023-11-23

**Authors:** Slaheddine Delleli, Ibrahim Ouergui, Christopher Garrett Ballmann, Hamdi Messaoudi, Khaled Trabelsi, Luca Paolo Ardigò, Hamdi Chtourou

**Affiliations:** ^1^High Institute of Sport and Physical Education of Sfax, University of Sfax, Sfax, Tunisia; ^2^Research Unit: Physical Activity, Sport and Health, UR18JS01, National Observatory of Sport, Tunis, Tunisia; ^3^High Institute of Sport and Physical Education of Kef, University of Jendouba, El Kef, Tunisia; ^4^Research Unit: Sports Science, Health and Movement, UR22JS01, University of Jendouba, El Kef, Tunisia; ^5^Department of Human Studies, University of Alabama at Birmingham, Birmingham, AL, United States; ^6^Research Laboratory: Education, Motricity, Sport and Health, EM2S, LR19JS01, University of Sfax, Sfax, Tunisia; ^7^Department of Teacher Education, NLA University College, Oslo, Norway

**Keywords:** music, ergogenic aid, warm-up, motivation, exercise

## Abstract

This systematic review summarized the studies that examined the pre-task music effects on performance aspects and quantitatively analyzed their outcomes. A systematic search for controlled studies investigating the acute effects of pre-task music on physical performance, cognitive aspects and associated psycho-physiological responses was performed through Scopus, PubMed, Web of Science and Cochrane Library databases up to 17 May 2023, with thirty studies fulfilled the inclusion criteria. Data was analyzed using the robust multilevel meta-analysis model of standardized mean difference “SMD” with 95% confidence intervals (95%CI) and prediction intervals (PI) were reported. Pre-task music induced improvements of completion time (SMD = −0.24; 95% CI = −0.46 to −0.01; PI = −0.82 to 0.35; *p* = 0.04), relative mean power (RMP) (SMD = 0.38; 95% CI = 0.16 to 0.60; PI = −0.36 to 1.12; *p* = 0.003) and fatigue (SMD = −0.20; 95% CI = −0.32 to −0.09; PI = −0.36 to −0.05; *p* = 0.01), moderate effects on relative peak power (RPP) (SMD = 0.53; 95% CI = 0.21 to 0.85; PI = −0.42 to 1.48; *p* = 0.005), and high effect on feeling scale (FS) (SMD = 2.42; 95% CI = 0.52 to 4.31; PI = −11.43 to 16.26; *p* = 0.03). Greater benefits were recorded in jumping performance in males than females (*p* = 0.01), and for active than trained subjects for completion time (*p* = 0.02), RPP (*p* = 0.02) and RMP (*p* = 0.03). Larger benefits were obtained for FS post-warming up than after testing (*p* = 0.04). Self-selected music induced greater effects than pseudo- and pre-selected for performance decrement index (*p* = 0.05) and FS (*p* = 0.02). It could be concluded that pre-task music improved psychological responses and fatigue-related symptoms associated with exercise performance enhancement.

## 1 Introduction

The ergogenic effects of listening to music on performance have been well documented and were highly relevant to both practitioners and those operating in the sport domain (Terry et al., 2020). The effectiveness of listening to music has been mainly attributed to its benefits on the psychological state and fatigue-related symptoms associated with exercise performance and recovery status improvement (Karageorghis and Priest, 2012; Bigliassi, 2015). Regarding sport performance, music interventions may serve to enhance psycho-physiological responses and recovery both during and after exercise (Ka-Lok et al., 2020). However, practically, athletes may not readily be able to listen to music during competition and thus must listen to music before training/competition to modulate mental and perceptual issues (i.e., relief of stressors and competition-related anxiety) (Karageorghis et al., 2021). This pre-event process can help athletes to be prepared for the game through rehearsing association between imaging and relaxation skills (Gavanda et al., 2022). It has been demonstrated that pre-task music intervention improved power and strength (Smirmaul, 2017), distracted the intentional focus from discomfort in order to reduce the perceived exertion (RPE) and improved physical performance (Terry et al., 2020). However, inconsistent results about pre-task music effects have been reported (Smirmaul, 2017), with some studies showing improved power output (Jarraya et al., 2012; Chtourou et al., 2012a, 2017), running speed (Tounsi et al., 2019; Jebabli et al., 2022), kicking performances (Ouergui et al., 2023a,b) and reduced performance decrement index (PDI) (Puad et al., 2020; Jebabli et al., 2023; Ouergui et al., 2023a), while others (Bigliassi et al., 2012, 2015; Loizou and Karageorghis, 2015; Gavanda et al., 2022) did not detect significant benefits even when music was self-selected (Fox et al., 2019). While there is no clear consensus about the underlying mechanisms related to performance changes after pre-task or warm-up music, collective evidence has suggested that motivation is a primary factor limiting the efficacy of pre-task and warm-up music (Smirmaul et al., 2014; Ballmann et al., 2019; Karow et al., 2020; Ballmann, 2021; Ballmann et al., 2021). This could implicate that various psychological factors largely affect subsequent performance and determine music intervention efficacy (Ballmann et al., 2021). To explain inconsistencies, psycho-physiological factors could be differentially altered by intrinsic features of music (i.e., tempo, genre, intensity, exposure duration, synchronization), experimental procedures (i.e., selection process, intensity and duration of exercise) and the listener’s characteristics (i.e., age, sex, training status) (Terry et al., 2020).

A recent meta-analysis about the effects of music in exercise and sports settings summarized data from pre, during and post-tasks interventions, resulting in high heterogeneous findings and limited their application (Terry et al., 2020). In fact, music interventions ‘timing is a moderating factor which could limit the generalization of the aforementioned meta-analysis findings (Bigliassi et al., 2012; Jebabli et al., 2022), as sports governing bodies forbid electronic devices’ use (e.g., personal music devices) during most game and competition scenarios (Chtourou et al., 2012b). Moreover, due to the increased body of evidence regarding the pre-task music effects on subsequent exercise responses with inconsistent findings being reported, conducting a meta-analytic review is highly pertinent in determining sound conclusions. Therefore, this systematic review and meta-analytic study was designed to summarize reports about pre-task music effects on exercise performance and related responses, meta-analyze their data taking into consideration the different moderators that could explain the variability across studies. Moreover, the study tends to highlight the limits of the existing literature and draw some practical recommendations that could improve the effectiveness of using pre-task music stimulation.

## 2 Materials and methods

This systematic review was conducted following the 2020 Preferred Reporting Items for Systematic Reviews and Meta-Analyses (PRISMA) (Page et al., 2021) and the PRISMA implemented in Exercise, Rehabilitation, Sport medicine and SporTs science (PERSiST) guidelines (Ardern et al., 2022). The protocol for this systematic review was published in the Open Science platform (OSF) on July 7, 2023. Registration DOI:10.17605/OSF.IO/XHBN4.

### 2.1 Search strategy

PubMed/MEDLINE, Scopus, Web of Science, Sport Discus, and Cochrane Library were searched without date limits or filters. The search was performed up to 17 May 2023 connecting the terms using the Boolean operators ‘AND’ and ‘OR’ with medical subject heading (MeSH) terms appropriately used. The search strategy on each database was presented in the Supplementary Table S1. The literature search was expanded further by hand searching review articles on the topic and by reference lists’ searching from the articles retrieved for relevant references.

### 2.2 Inclusion and exclusion criteria

The PICO (i.e., population, intervention, comparator, and outcome) model was used in studies inclusion:

- Population: Healthy subjects.
- Intervention: Acute effects of isolated music intervention during warm-up or before exercise.
- Comparator: No music stimulus (control).
- Outcomes: Any physiological, affective, or psychophysical responses associated with an objective performance outcome (i.e., time, distance, speed, power, repetitions, etc.) or cognitive aspects.

These eligibility criteria resulted in excluding studies that were not associated with the established PICO criteria. Additionally, studies were excluded if music intervention effect could not be isolated from other stimulation (e.g., accompanying video footage, or brainwave synchronizer), if a case study or a longitudinal study design have been used, if a clinical or special population has been studied, or if a subjective measure of performance was used (e.g., interview). Moreover, books, citations, trial registry records, conference proceedings, systematic reviews, and narrative reviews were excluded.

### 2.3 Study selection

The retrieved articles were first assessed for duplication using the software “Endnote 20” (Camelot UK Bidco Limited-Clarivate, United Kingdom) before being considered for inclusion. After duplicate removal, relevant articles’ titles review was conducted before examining articles’ abstracts and then fully-published peer-reviewed articles. Two reviewers conducted the process independently and consensus was used to resolve disagreements between them. The reasons for the exclusion of full-text articles were recorded.

### 2.4 Data extraction

For studies meeting inclusion criteria, data were summarized in a Microsoft Excel spreadsheet. A piloted data extraction form with the following items: author(s), year of publication, sample size and sex, sample age, exercise performed, main outcomes, exposure duration, music selection, music tempo, loudness and the main results were used. For the main results, all data about the effect of pre-task music on physical performance, perceived exertion, fatigue symptoms, affective and physiological responses before, during or/and following exercise were extracted from the papers. All eligible outcomes including repeated measurements at different time points were considered as one unit of evidence and coded accordingly. If data were not reported in a way that was conducive to extraction for our analysis, we request appropriate data from respective authors [i.e., mean (standard deviation), raw data]. If authors did not respond our request, we extracted relevant data in studies that only reported graphical information (Drevon et al., 2017) using WebPlot Digitizer (v4.3, Ankit Rohatgi). The data extraction process was double-checked by a second reviewer to avoid any selection bias and data extraction flaws.

### 2.5 Moderator variables coding

A wide variety of moderating factors were tested and coded as presented in Table 1. The included studies were coded for music characteristics (i.e., selection, tempo, exposure duration), study characteristics (i.e., time of experimentation, timing of variable measurement, test duration), and participant characteristics (i.e., sex, age, and training status).

**Table 1:** Moderating factors and their codes.

Moderator	Criteria
*Music characteristics*	
Music selection	Self-selected: music stimuli selected by participants
	Pre-selected: music stimuli selected by the experimenter(s)
	Pseudo: music stimuli selected by participants from an experimenter-defined list or the opposite
Music tempo	Fast: refers to music with a tempo >120 bpm
	Slow-to-medium tempo: refers to music with a tempo <120 bpm
Exposure duration	Kept as a continuous moderator
*Participants characteristics*	
Training status	Trained: refers to participants who engaged in regular physical activity (3 times/week)
	Untrained/active: refers to participants for whom physical activity was not habitual or irregular
Sex	Male
	Female
	Mixed/unspecified
Age	Kept as a continuous moderator
*Study characteristics*	
Time of day of experimentation	Morning
	Evening
	Unspecified
Timing of variable measurement	Before exercise
	During exercise
	After exercise
Test duration	Short duration: ≤2 min
	Long duration: >2 min
	Exhaustive: Exercise to failure

### 2.6 Assessment of risk of bias

The methodological quality of the included studies was assessed using the Physiotherapy Evidence Database (PEDro) scale (Maher et al., 2003; de Morton, 2009). Given that blinding in the traditional experimental sense is not possible with a music treatment (Terry et al., 2020), items 5–7 (which are specific to blinding)were removed from the scale (Garbisu-Hualde and Santos-Concejero, 2021). After these items’ removal, the maximum result on the modified PEDro scale was 7 (i.e., the first item is not included in the final score)and the lowest was 0 (Garbisu-Hualde and Santos-Concejero, 2021). The quality assessment was interpreted as follows: poor quality (≤3 points), moderate quality (4–5 points), and high quality (6–7 points). Two reviewers performed the methodological quality assessment independently.

### 2.7 Data analysis

#### 2.7.1 Effect sizes calculation

The standardized mean difference “SMD” was calculated from primary studies by subtracting the means and SDs from music and no music conditions, the sample size and the raw correlation coefficient. For within-participant effects, pre-post correlations for measures have not been reported, therefore, we adopted a range of values for correlation coefficients (*r* = 0.5, 0.7, and 0.9) and examined the sensitivity of the results to each of these values. Since the overall results were relatively insensitive to this range, we reported the results for *r* = 0.7 here and included the results for the other assumed correlation coefficients in the Supplementary File.

#### 2.7.2 Overall effects synthesis

The effect sizes’ independence is crucial to avoid an information overlap and to provide unbiased effect size’s estimations (Cheung, 2014; Gucciardi et al., 2021). Multilevel meta-analysis has been reported as a valid and efficient way to handle dependent effect sizes within a study (Assink and Wibbelink, 2016; Moeyaert et al., 2017). Most studies in the present systematic review are multi-arms. Therefore, to deal with the statistical dependency caused by multiple observations inclusion from the same study, a multi-level, random-effects model for meta-analysis was performed using the “metaphor” package (rma.mv function) (Viechtbauer, 2010) and complemented by a cluster-robust inference method (Pustejovsky and Tipton, 2022) to generate an overall mean effect size (ES) and 95% confidence interval (95%CI). Furthermore, 95% prediction interval (PI) was calculated since it serves to determine the expected range of ES in future primary studies (IntHout et al., 2016). A multilevel meta-analysis was conducted for every outcome separately and model parameters were estimated by the restricted maximum likelihood estimation method.

#### 2.7.3 Heterogeneity and moderators’ analysis

To calculate heterogeneity across studies, Q statistic and *I*^2^ were used (Higgins et al., 2003). Heterogeneity was indicated if the Q statistic reached a significance level of *p* < 0.050 (Hedges and Olkin, 2014). *I*^2^ values indicate heterogeneity degree of effects as follows: 0–40% indicates no heterogeneity, 30–60% moderate heterogeneity, 50–90% substantial heterogeneity, and 75–100% considerable heterogeneity (Higgins et al., 2019).

Exploratory subgroup comparisons of moderator variables were performed, including music selection, tempo, sex, training status, timing of variable measurement, test duration, and time of day. Meta-regressions were calculated for mean age, and exposure duration to the music. Moderator analysis was performed when there were sufficient observations per group (i.e., at least 3 observations) (Spruit et al., 2016) and robust estimates were produced.

#### 2.7.4 Publication bias

The risk of small study bias was visualized through contour-enhanced funnel plots. Moreover, multilevel extension of the Egger’s test was used to detect publication bias. The variance of effects was converted into standard error and the moderation model was performed (Fernández-Castilla et al., 2021). The robust estimates results of the Egger’s test were produced for all analyses.

#### 2.7.5 Outlier detection

For all meta-analytic models, outlier and influential case diagnostics were performed by calculating Cook’s distance and standardized residuals, respectively (Cook, 1977; Viechtbauer and Cheung, 2010). Cases were considered outliers when their Cook’s distance’s values greater three times than their respective mean, and with a standardized residual value greater than 3, in absolute values (Gucciardi et al., 2021). In the case of existing outliers, the overall effects were recalculated to assess the robustness of the fitted model.

## 3 Results

### 3.1 Search results

**Figure 1:**
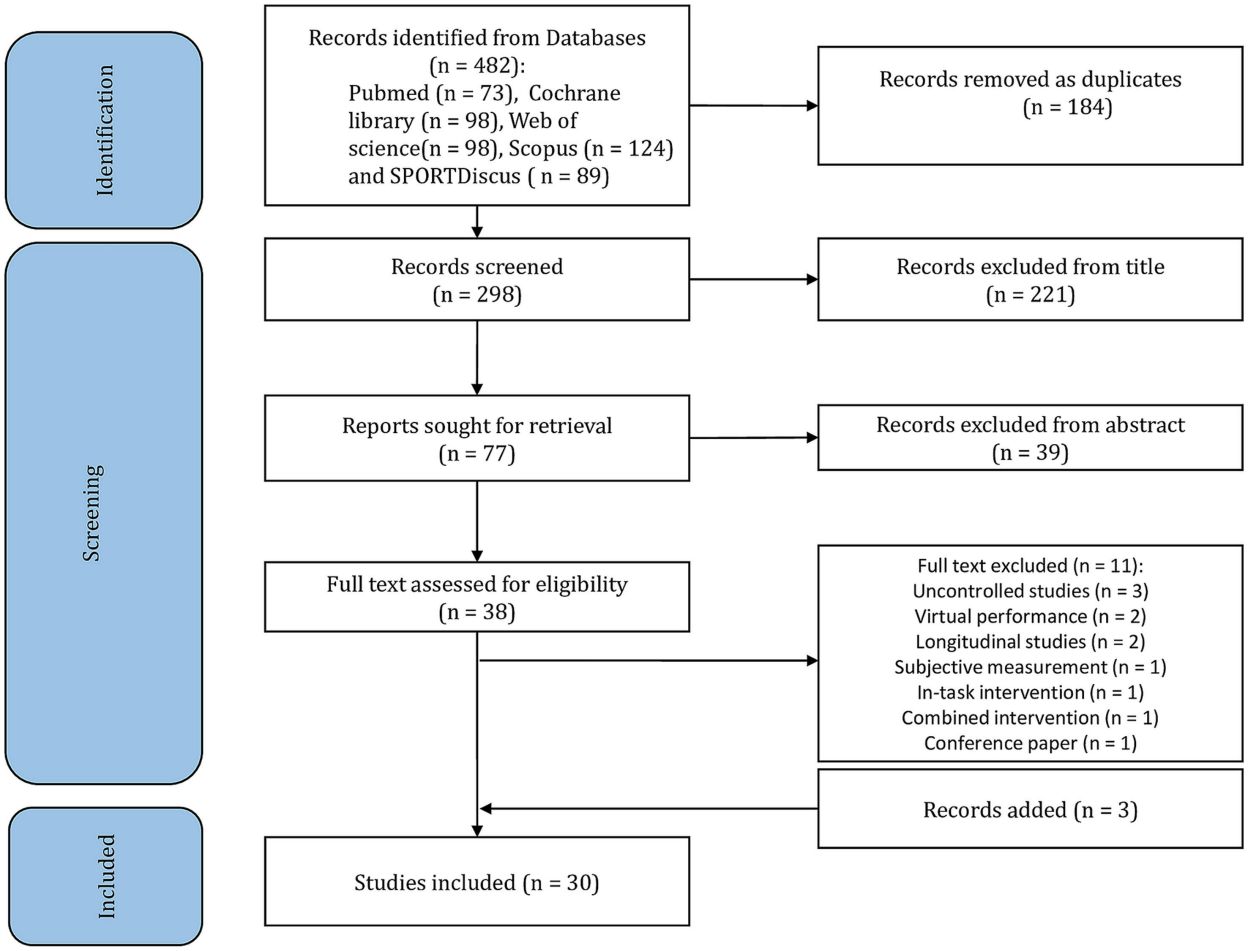
Flow diagram of the search process.

A summary of the search process is shown in Figure 1. Search strategies identified 482 citations related to music in physical activity and exercise. After 184 duplicates’ removal, title and abstract of 298 citations were screened and 38 studies were targeted for detailed review (i.e., 260 excluded). In total, 11 studies which did not meet all inclusion criteria were excluded after full-text screening. The excluded studies and exclusion reasons were listed in the Supplementary Table S2. After an additional search on Google scholar and in the reference lists of the relevant studies, three published articles were added as relevant papers, resulting in a net number of 30 studies from the overall search process.

### 3.2 Study characteristics

The 30 included studies recruited 474 subjects divided into 360 males, 80 females and 34 with unspecified sex. Sample sex was exclusively females in one study (Ghazel et al., 2022), mixed in seven studies (Eliakim et al., 2007; Bishop et al., 2013; Fox et al., 2019; Tounsi et al., 2019; Karow et al., 2020; Ouergui et al., 2023a,b), and exclusively males in the remaining studies, excepting two studies (Bigliassi et al., 2012; Puad et al., 2020) where sample sex was unspecified. The sample size recruited in the included studies varied from 9 to 33. Participants were either adolescents (i.e., <18 years) or young adults (i.e., 30<age > 18 years) with a mean age ranged from 16.4 (0.3) to 26.04 (2.98) years.

All the studies were multi-armed and reported multiple measurement points through different times of day (Chtourou et al., 2012a; Belkhir et al., 2019; Jarraya and Jarraya, 2019; Belkhir et al., 2020, 2021; Khemila et al., 2021; Bayrakdaroğlu et al., 2022), menstrual cycle phases in females (Ghazel et al., 2022), music timing application (Bigliassi et al., 2012; Jebabli et al., 2022, 2023), music intrinsic components (e.g., tempo and volume) (Bishop et al., 2013; Belkhir et al., 2020, 2021; Gavanda et al., 2022; Ouergui et al., 2023a,b), sample sex and training background (Chtourou et al., 2017; Fox et al., 2019; Tounsi et al., 2019; Ouergui et al., 2023a,b) and selection process (Belkhir et al., 2019, 2020, 2021; Fox et al., 2019; Karow et al., 2020; Bentouati et al., 2022; Gavanda et al., 2022; Ouergui et al., 2023b). Among the included studies, 22 used selected music by the investigators (i.e., PSM), 10 used selected music by participants (i.e., SSM), and three used music selected within specific pre-determined lists (i.e., pseudo). Considering testing protocols, 10 studies (Eliakim et al., 2007; Jarraya et al., 2012; Chtourou et al., 2012a,b, 2017; Loizou and Karageorghis, 2015; Fox et al., 2019; Khemila et al., 2021; Bayrakdaroğlu et al., 2022; Bentouati et al., 2022) used 30 s Wingate test, four studies (Belkhir et al., 2020, 2021; Gavanda et al., 2022; Ghazel et al., 2022) used jumping tests, four others used repeated sprint tests (Tounsi et al., 2019; Puad et al., 2020; Ghazel et al., 2022; Jebabli et al., 2023), three researches (Ghazel et al., 2022; Ouergui et al., 2023a,b) used agility tests, two studies used the 5-m shuttle run test (Aloui et al., 2015; Belkhir et al., 2019), two studies used resistance exercises (Arazi et al., 2015; Ballmann et al., 2021), and two studies (Ouergui et al., 2023a,b) used sport-specific tests. The duration of pre-task music exposure varied from one to 15 min, with the most prevalent duration being 10 min (23 studies). The studies characteristics and the main results were presented in Table 2.

**Table 2:** Studies characteristics.

Study	Sample size and sex	Age (years)	Task	Outcome	Training status	Selection	Tempo (beats/min)	Exposure duration (min)	Volume (db)	Main results
Ballmann et al. (2021)	10 M	22.8 (5.8)	Bench press	MP, barbell velocity, repetitions to failure, motivation	Resistance trained	SSM (PREF) NM	131 (6)	3	Unspecified	↑Velocity, power output, repetitions to failure, and motivation
Belkhir et al. (2019)	12 M	21.82 (2.47)	5-m shuttle run test	HD, TD, FI, RPE, FS	Soccer players	SSM PSM NM	>120–140	10	Unspecified	↑HD and TD in the morning and afternoon ↓FS with SSM ↑RPE with SSM No effect on FI
Belkhir et al. (2021)	15 M	21.02 (1.52)	30-s continuous Jump test	Jump height, FI, FS	Soccer players	PSM SSM NM	60 (low) 120–140 (high)	10	Unspecified	↑Jump height and FS with PSM and SSM compared to NM condition at 07 and 17 h
Belkhir et al. (2020)	16 M	20.89 (1.51)	30-s continuous Jump	FI, jump height, RPE, FS	Trained	PSM (motivational vs. synchronous) NM	Synchronous (60) Motivational (>120–140)	10	Unspecified	↑Jump height_mean_ and jump height_max_ with music at both times of day ↑RPE with motivational than NM at both times of day FS ↑with motivational than NM in the morning No effect on FI
Bentouati et al. (2022)	10 M	20.42 (1.08)	30s Wingate test	PP, MP, RPE	Active	Pseudo (PREF vs. NPREF) NM	>120–140	10	Unspecified	↑PP with SSM ↓RPE with SSM compared to NM and NPM after warm-up and testing No effect on FI
Chtourou et al. (2012a)	12 M	22.4 (1.7)	30s Wingate test	PP, MP, RPE	Active	PSM NM	>120 to 140	10	Unspecified	↑PP and MP and RPE with music in the morning and evening
Chtourou et al. (2017)	24 M: 12 sprinters 12 long-distance runners	Sprinters [20.1 (1.6)], long-distance runners [21.2 (2.1)]	30s Wingate test	RPE, PP, MP, POMS	Trained	PSM NM	>120–140	10	75–80	↑PP, MP, RPE and vigor in sprinters
Chtourou et al. (2012b)	9 M	19.56 (1.88)	30s Wingate test	RPE, MP, PP, FI	Trained	PSM NM	120–140	10	Unspecified	↑MP and PP No effect on RPE and FI
Eliakim et al. (2007)	24 (12 M 12 M)	F: 16.4 (0.3) M: 17.0 (0.2)	30s Wingate test	PP, MP, FI, RPE, HR	Volleyball players	PSM NM	140	10	70% of the maximal volume	↑PP and HR mean No effect on MP and FI
Jarraya et al. (2012)	12 M	20.6 (1.8)	30s Wingate test	HR, RPE, MP, PP, FI	Trained	PSM NM	120–140	10	Unspecified	↑PP and MP No effect on HR, RPE and FI
Jarraya and Jarraya (2019)	14 M	17 ± 1.2	RT test, the barrage test, the trail-making test and the paper-folding test	RT, attention, executive functions and spatial memory skills	Tennis players	SSM NM	Unspecified	10	Unspecified	↑Cognitive parameters at morning and evening
Smirmaul et al. (2014)	18 M	26 (4)	200 m freestyle swimming	Motivation, FAS, RPE, FS, swimming time	Trained	SSM NM	118	5	Unspecified	↑Motivation, and ↓Swimming time No effect on RPE, FAS and FS
Bayrakdaroğlu et al. (2022)	17 M	21.53 (1.32)	30s Wingate test	PP, MP, RPP, RMP, FI	Futsal players	PSM NM	120–140	10	Unspecified	↑MP No effect on PP, RPP, RMP, and FI
Khemila et al. (2021)	12 M	20.3 (2.0)	30s Wingate test	MP, PP, SRT, CRT, selective attention, POMS, RPE	Active	PSM NM	>120 to 140	10	Unspecified	↑PP and MP in the morning ↓Post-exercise cortisol in the morning ↓RPE in the morning reduced the negative mood states ↓SRT and CRT in the evening
Jebabli et al. (2022)	25 M	21.0 (1.1)	6-min all-out exercise test	Mean running speed, covered distance, HR_peak_, HR_mean_, [bLa^−^], RPE, FS	Active	SSM (PREF) NM	130 (10)	10	70	↑Running speed and covered distance No effect on HR_peak_, HR_mean_, [bLa^−^], RPE, and FS
Jebabli et al. (2023)	19 M	22.1 (1.2)	2 sets of 5*20-m repeated-sprints	Total time, best time, [bLa], HR, RPE, and FS	Sport-science students	SSM (PREF) NM	> 140	10	70	↓Total time, fast time index and FI in set 1 ↑[bLa] No effects on HR, RPE, and FS
Aloui et al. (2015)	9 M	21 (1.1)	5-m multiple shuttle run test	Covered distance, PACES, anxiety, self confidence	Active	SSM NM	120–140	10	Unspecified	↓Anxiety No effect on covered distance, PACES, and self confidence
Karow et al. (2020)	12 (6 M, 6F)	21.1 (1)	2000-m rowing	RPP, trial time, HR, RPE and motivation	Active	Pseudo (PREF vs. NPREF) NM	>120	5	Unspecified	PREF music ↑RPP, completion time, HR, and motivation No effect on RPE
Arazi et al. (2015)	12 M	24 (2)	Circuit-type resistance exercise	RPE, HR, completion time	Trained	PSM NM	130	10	Unspecified	↓Completion time No effect on RPE, HR
Ghazel et al. (2022)	14 F	22.2 (0.9)	SJ, CMJ, agility T test, RSA	Jump height, agility time, sprint time, RPE, POMS	Handball players	PSM NM	140	10	Unspecified	↑SJ in the different MCP ↓RPE at the end of the RSA test No effects on CMJ, agility and RSA.
Bigliassi et al. (2012)	10 (Unspecified)	24 (1)	Maximal incremental test (MIT) and three 5-km time trials	Brunel Mood Scale, RPE, PP, RPP, mean speed, MP, total time	Cyclists	SSM NM	Unspecified	10	Unspecified	↓RPE No effect on completion time, mean speed, PP, MP and mood sate
Bigliassi et al. (2015)	15 M	24.87 (2.47)	5-km run	During (performance time, HR, and RPE), and after (mood, RPE, and HRV)	Long-distance runners	SSM NM	129.9 (7.33)	10	75 (5)	No effect on completion time, HR, mood or RPE
Bishop et al. (2013)	12 (6 M, 6F)	21.2 (3)	Three-choice reaction time task	Brain responses during reactive task performance	Tennis players	PSM NM	99 129 161	1.5	5575	Faster tempi and higher intensity yielded activation in structures integral to visual perception, allocation of attention, and motor control.
Gavanda et al. (2022)	13 M	25.5 (2.6)	Five squat jumps	Jump height, jump power, mood states	Volleyball players	PSM (relaxing) SSM (stimulating) NM	Relaxing: 70 (0) Stimulating: 124.7 (19.4)	1	Unspecified	↑Moods components No effect on Jump height and power
Puad et al. (2020)	24 (unspecified)	26.04 (2.98)	RAST	HR, RPE, PP, MP, FI	Trained	PSM NM	>120	10	Unspecified	↑HR and ↓FI No effect on PP, MP and RPE
Tounsi et al. (2019)	33 (19 M 14F)	17 (0.3) 17 (0.2)	RSA	Best sprint time, mean sprint time, RSA, FI, AL	Soccer players	PSM NM	>130–140	15	Unspecified	↑Best and mean sprint times in females No effect of condition on AL and FI
Fox et al. (2019)	16 (8 M, 8F)	23.6 (4.8)	30s Wingate test	MP, PP, RMP, RPP, RPE, FI	Active	SSM PSM NM	119.7 (20.97)	10	Self-controlled	No effect on PP, MP, RPP, RMP, FI and RPE
Ouergui et al. (2023a)	20 (10 M, 10F)	17.5 (0.7)	TSAT, FSKT-10s, FSKT-mult	Agility time; number of techniques; FI; RPE, PACES	Taekwondo athletes	PSM NM	140 200	10	80 60	140 beats.min^−1^ + 80 dB ↑Agility time; number of techniques; and ↓FI ↑RPE ↑PACES in 140 beats.min^−1^ + 80 dB condition
Ouergui et al. (2023b)	20 (10 M, 10F)	17.7 (0.82)	TSAT, FSKT-10s, FSKT-mult	agility time; number of techniques; FI; RPE, PACES	Taekwondo athletes	SSM (PREF) Pseudo (NPREF) NM	130 (8)	10	60 80	SSM: ↓ RPE and FI ↑Agility time; number of techniques
Loizou and Karageorghis (2015)	15 M	26.3 (2.8)	30s Wingate test	PP, MP, FI, HRV, and affective scale	Trained	PSM (Music-only) NM	Unspecified	5	75	No effects on the Wingate outcomes, affective state and HRV.

AL, affective load; PP, peak power; MP, mean power; RPP, relative peak power; RMP, relative mean power, FI, fatigue index; FS, feeling scale; RPE, rate of perceived exertion; PACES, physical enjoyment; HR, heart rate; HRV, heart rate variability; RSA, repeated sprint ability test; HD, higher distance; TD, total distance; RT, reaction time; SRT, simple reaction time; CRT, choice reaction time; TSAT, taekwondo specific agility test; FSKT-10s, 10 s frequency speed of kick test; FSKT-mult, multiple frequency speed of kick test; SJ, squat jump; CMJ, counter movement jump; POMS, profile of mood state; [bLa], blood lactate concentration; MCP, menstrual cycle phases; SSM, self-selected music; PSM, pre-selected music; NM, no music; PREF, preferred; NPREF, non-preferred; ↑, improve; ↓, reduce.

### 3.3 Methodological quality

By removing blinding criteria, included studies presented a moderate to high methodological qualities (5–7 points) as presented in Table 3. The primary issue with the Pedro items is the randomization procedure, which was not covered in depth in the included researches.

**Table 3:** Pedro scale items (I) and studies’ scores.

Study	I1	I2	I3	I4	I8	I9	I10	I11	Total
Ballmann et al. (2021)	Yes	-	+	+	+	+	+	+	6
Belkhir et al. (2019)	Yes	+	+	+	+	+	+	+	7
Belkhir et al. (2021)	Yes	+	+	+	+	+	+	+	7
Belkhir et al. (2020)	Yes	+	+	+	+	+	+	+	7
Bentouati et al. (2022)	Yes	+	+	+	+	+	+	+	7
Bishop et al. (2013)	No	+	-	+	+	+	+	+	6
Chtourou et al. (2012a)	No	-	-	+	+	+	+	+	5
Chtourou et al. (2017)	No	+	+	+	+	+	+	+	7
Chtourou et al. (2012b)	No	-	-	+	+	+	+	+	5
Eliakim et al. (2007)	No	+	-	+	+	+	+	+	6
Jarraya et al. (2012)	No	-	+	+	+	+	+	+	6
Jarraya and Jarraya (2019)	No	+	-	+	+	+	+	+	6
Smirmaul et al. (2014)	No	+	+	+	+	+	+	+	7
Bayrakdaroğlu et al. (2022)	Yes	-	+	+	+	+	+	+	6
Khemila et al. (2021)	Yes	+	-	+	+	+	+	+	6
Jebabli et al. (2022)	Yes	+	+	+	+	+	+	+	7
Jebabli et al. (2023)	Yes	+	+	+	+	+	+	+	7
Aloui et al. (2015)	No	+	-	+	+	+	+	+	6
Karow et al. (2020)	No	-	+	+	+	+	+	+	6
Loizou and Karageorghis (2015)	yes	-	-	+	+	+	+	+	5
Arazi et al. (2015)	yes	+	-	+	+	+	+	+	6
Ghazel et al. (2022)	Yes	+	+	+	+	+	+	+	7
Bigliassi et al. (2012)	Yes	-	-	+	+	+	+	+	7
Bigliassi et al. (2015)	Yes	+	-	+	+	+	+	+	7
Gavanda et al. (2022)	Yes	-	+	+	-	+	+	+	5
Puad et al. (2020)	No	-	+	+	+	+	+	+	6
Tounsi et al. (2019)	No	+	-	+	+	+	+	+	6
Fox et al. (2019)	No	-	-	+	+	+	+	+	5
Ouergui et al. (2023a)	Yes	+	-	+	+	+	+	+	6
Ouergui et al. (2023b)	Yes	+	-	+	+	+	+	+	6

I1, participants eligibility; I2, random allocation; I3, concealed allocation; I4, groups similar at baseline; I8, less than 15% dropouts; I9, intention-to-treat analysis; I10, between-group statistical comparisons; I11, point measures and variability data.

### 3.4 Meta-analysis

#### 3.4.1 Completion time

Pre-task music was associated with a small significant improvement for completion time [SMD = −0.24; 95%CI = −0.46 to −0.01; PI = −0.82 to 0.35; *p* = 0.04] with moderate heterogeneity (Q_30_ = 112.85; *p* < 0.001; *I*^2^ = 47.28%). Moderators’ analysis showed that training status was a significant factor affecting completion time (F_2,1.56 =_ 121.23, *p* = 0.02), with greater effects on moderately active (SMD = −0.30; 95%CI: −0.50, −0.11) than highly trained subjects (SMD = −0.21; 95%CI: −0.53, 0.10) (Supplementary Table S3).

Funnel plot of per-study standard error by SMD (Supplementary Figure S1) indicated significant asymmetry. The robust multilevel model of Egger’s test showed potential risk of publication bias (F_1, 1.39_ = 228.27, *p* = 0.02).

Outlier, and influential case diagnostics were performed and showed that excluding outlier residuals (SMD = −0.23; 95%CI = −0.44 to −0.02; PI = −0.78 to 0.32; *p* = 0.04; *I*^2^ = 45.82%) and Cook’s outliers (SMD = −0.32; 95%CI = −0.55 to −0.09; PI = −0.86 to 0.21; *p* = 0.01; *I*^2^ = 37.74%) did not affect the magnitude effect, heterogeneity, and significance level.

#### 3.4.2 Performance decrement index (PDI)

Pre-task music was associated with non-significant benefits for PDI [SMD = −0.39; 95% CI = −0.92 to 0.14; PI = −2.90 to 2.12; *p* = 0.14] with considerable heterogeneity (Q_49_ = 419.69; *p* < 0.001; *I*^2^ = 94.90%). Music selection significantly moderated PDI (*F*_3,1.78_ = 23.44; *p* = 0.05), with greater benefit from SSM (SMD = −1.35; 95%CI: −2.94, 0.24) than pseudo selected music (SMD = 0.50; 95%CI = −1.37 to 2.37) and PSM (SMD = −0.34; 95%CI = −0.93 to 0.24) (Supplementary Table S4).

Funnel plot of per-study standard error by SMD (Supplementary Figure S2) indicated significant asymmetry. The robust multilevel model of Egger’s test showed a potential risk of publication bias (*F*_1,2.02_ = 45.18; *p* = 0.02).

Outlier, and influential case diagnostics were performed and showed that excluding outlier residuals (SMD = −0.36; 95%CI = −0.88 to 0.15; PI = −2.77 to 2.04; *p* = 0.15; *I*^2^ = 94.57%) and Cook’ s outliers (SMD = −0.17; 95%CI = −0.58 to 0.23; PI = −2.00 to 1.65; *p* = 0.37; *I*^2^ = 90.93%) reduced the magnitude effect, without affecting the significance level, and heterogeneity.

#### 3.4.3 Relative peak power

Pre-task music was associated with a significant benefit for RPP [SMD = 0.53; 95% CI = 0.21 to 0.85; PI = −0.42 to 1.48; *p* = 0.005] with moderate heterogeneity (Q_17_ = 56.17; *p* < 0.001; *I*^2^ = 69.90%). Training status moderated RMP (F_2,4.61_ = 9.30; *p* = 0.02), with greater effect on physically active (SMD = 0.63; 95%CI: −0.41, 1.68) than highly trained subjects (SMD = 0.45; 95%CI: 0.19, 0.72) (Supplementary Table S5).

Funnel plot of per-study standard error by SMD (Supplementary Figure S3) indicated significant asymmetry. The robust multilevel model of Egger’s test showed a potential risk of publication bias (*F*_1,2.18_ = 68.73; *p* = 0.01).

Outlier and influential case diagnostics were performed and showed no existing outlier residuals and that excluding Cook’s outliers (SMD = 0.57; 95%CI = 0.23 to 0.92; PI = −0.42 to 1.56; *p* = 0.005; *I*^2^ = 69.74%) did not affect the magnitude effect, significance level, and heterogeneity.

#### 3.4.4 Relative mean power

Pre-task music was associated with a significant benefit for RMP [SMD = 0.38; 95% CI = 0.16 to 0.60; PI = −0.36 to 1.12; *p* = 0.003] with moderate heterogeneity (Q_20_ = 55.22; *p* < 0.001; *I*^2^ = 62.64%). Training status moderated significantly RMP (*F*_2,6.05_ = 6.72; *p* = 0.03), with greater effects in physically active (SMD = 0.46; 95%CI: −0.07, 0.98) than highly trained subjects (SMD = 0.31; 95%CI: 0.07, 0.56) (Supplementary Table S6).

Funnel plot of per-study standard error by SMD (Supplementary Figure S4) indicated significant asymmetry. Furthermore, the robust multilevel model of Egger’s test showed a potential risk of publication bias (*F*_1,2.25_ = 46.02; *p* = 0.02).

Outlier, and influential case diagnostics were performed and showed no existing outlier residuals and that excluding Cook’s outliers (SMD = 0.32; 95%CI = 0.10 to 0.54; PI = −0.31 to 0.95; *p* = 0.008; *I*^2^ = 54.77%) did not affect the magnitude effect, significance level, and reduced heterogeneity,

#### 3.4.5 Jump height

Pre-task music was associated with a non-significant improvement for jump height [SMD = 2.24; 95% CI = −1.74 to 6.23; PI = −7.04 to 11.53; *p* = 0.17] with considerable heterogeneity (Q_15_ = 202.33; *p* < 0.001; *I*^2^ = 98.78%). Sex significantly moderated jump height (F_2,1_ = 3649.66; *p* = 0.012), with greater effects in males (SMD = 3.04, 95% CI = −3.13 to 9.21) than females (SMD = 0.10, 95%CI = 0.09–0.11) (Supplementary Table S7).

Funnel plot of per-study standard error by SMD (Supplementary Figure S5) indicated significant asymmetry. The robust multilevel model of Egger’s test showed potential risk of publication bias (*F*_1, 1.16_ = 1380.76; *p* = 0.01).

Outlier and influential case diagnostics were performed and showed no outlier residuals and that excluding Cook’s outliers (SMD = 1.51; 95%CI = −1.12 to 4.15; PI = −4.26 to 7.28; *p* = 0.17; *I*^2^ = 97.41%) reduced the magnitude effect, without affecting heterogeneity, and the significance level.

#### 3.4.6 Ratings of perceived exertion

Pre-task music was associated with non-significant benefit for RPE [SMD = 0.01; 95% CI = −0.26 to 0.28; PI = −1.59 to 1.60; *p* = 0.96] with substantial heterogeneity (Q_104_ = 574.29; *p* < 0.001; *I*^2^ = 89.22%). Moderators’ analysis (Supplementary Table S8) showed no moderator to affect RPE.

Funnel plot of per-study standard error by SMD (Supplementary Figure S6) indicated no significant asymmetry. The robust multilevel model of Egger’s test showed no potential risk of publication bias (*F*_1,4.22_ = 3.45; *p* = 0.13).

Outlier and influential case diagnostics were performed and showed that excluding outlier residuals (SMD = -0.06; 95%CI = −0.30 to 0.19; PI = −1.43 to 1.32; *p* = 0.63; *I*^2^ = 86.33%) and Cook’s outliers (SMD = −0.09; 95%CI = −0.31 to 0.13; PI = −1.26 to 1.08; *p* = 0.39; *I*^2^ = 81.81%) did not affect the magnitude effect, significance level, and heterogeneity.

#### 3.4.7 Feeling scale

Pre-task music was associated with high significant benefit for FS [SMD = 2.42; 95% CI = 0.52 to 4.31; PI = −11.43 to 16.26; *p* = 0.03] with considerable heterogeneity (Q_23_ = 439.18, *p* < 0.0001; *I*^2^ = 99.39%). Music selection significantly affected FS (F_2,1.78_ = 66.57; *p* = 0.02), with higher effect of SSM (SMD = 2.68; 95%CI: −5.78, 11.14) than PSM (SMD = 2.35; 95%CI: 1.36, 3.34) (Supplementary Table S9). Moreover, timing of variable measurement was an affective factor (*F*_2,1.79_ = 32.57; *p* = 0.04), with higher effects post warm-up (SMD = 4.65; 95%CI: −1.80, 11.10) than after testing (SMD = −0.26; 95%CI: −6.37, 5.86). In addition, training status was a moderator factor (*F*_2, 1.03_ = 596.13; *p* = 0.03), with greater effect on trained (SMD = 2.77; 95%CI:0.12, 5.41) than active subjects (SMD = 0.15; 95%CI: 0.11, 0.19).

Funnel plot of per-study standard error by SMD (Supplementary Figure S7) indicated significant asymmetry. The robust multilevel model of Egger’s test showed potential risk of publication bias (*F*_1,1.67_ = 495.64; *p* = 0.005),

Outlier, and influential case diagnostics were performed and showed that excluding outlier residuals (SMD = 1.98; 95%CI = −0.32 to 4.28; PI = −10.25 to 14.20; *p* = 0.07; *I*^2^ = 99.26%)and Cook’s outliers (SMD = 1.98; 95%CI = −0.32 to 4.28; PI = −10.25 to 14.20; *p* = 0.07; *I*^2^ = 99.26%) reduced the magnitude effect, without affecting heterogeneity, and the significance level.

#### 3.4.8 Fatigue symptoms

Pre-task music was associated with significant benefit for fatigue symptoms [SMD = −0.20; 95% CI = −0.32 to −0.09; PI = −0.36 to −0.05; *p* = 0.01] with low heterogeneity (*Q*_7_ = 8.33; *p* = 0.30; *I*^2^ = 2.19%). Moderators’ analysis (Supplementary Table S10) showed no moderators to affect fatigue symptoms.

Funnel plots of per-study standard error by SMD (Supplementary Figure S8) indicated no significant asymmetry. The robust multilevel model of Egger’s test showed no potential risk of publication bias (*F*_1, 3_ = 5.16; *p* = 0.11).

Outlier and influential case diagnostics were performed and showed no outlier residuals or Cook’s outliers.

## 4 Discussion

This is a meta-analytic review about pre-task music effects on physical performances and the associated psycho-physiological aspects. The meta-analysis showed that pre-task music was associated with significant improvement in completion time, relative mean and peak powers, affective valence, and fatigue symptoms. Moderators’ analysis revealed that sex was a significant factor that affected jump height, with greater performance in males than females. Training status modulated the physical performance with completion time, RPP and RMP were greater in active than trained subjects. In addition, variable measurement timing moderated pre-task music effect on FS with larger benefits post-warming up than after testing. Furthermore, music selection process was a significant moderator of PDI and FS with greater effects from SSM than PSM and pseudo selected music.

### 4.1 Main effects

The present study showed that pre-task music improved different aspects of both physical and psychological aspects of responses to exercise. The findings of the present systematic review with meta-analysis supported previous meta-analyses which suggested that music was associated with significant beneficial effects for physical performance (Terry et al., 2020) and that when outcomes were expressed relatively to body mass (i.e., RPP and RMP), listening to music induced significant moderate-sized effects (Castaneda-Babarro et al., 2020). According to Karageorghis and Terry (2011), listening to the appropriate music may boost exercise performance up to 15%, which is comparable to commonly used supplemental ergogenic aids (Karageorghis and Terry, 2011). It has been shown that music acts as an ergogenic aid which improves exercise performance by attenuating exhaustion or increasing work capacity (Karageorghis and Priest, 2012). A decrease in power output over the course of total effort time during a task reflects a fatigue index (Castaneda-Babarro et al., 2020). In the present meta-analysis, the effect of pre-task music on PDI was moderate but non-significant. The observed effect could be practically important in sport settings since seemingly trivial performance improvements for an athlete may be decisive for the event’s outcome (Hopkins et al., 1999). The moderate PDI decrease might be explained by the energy economy improvement (Atkinson et al., 2004). However, it is crucial to keep in mind that PDI largely depends on the muscle fibers’ type of participants, as a larger percentage of muscle fibers enable higher peak power but a higher fatigue rate (Castaneda-Babarro et al., 2020). Since music stimulates the motor cortex and other regions of the brain involved in the control of movement, its effects on power output may be related to changes in neuromuscular activity (Gordon et al., 2018). This could be evident since pre-task music has showed a likely ergogenic effect on short and predominantly anaerobic tasks (Smirmaul, 2017). Physiologically, warm-up music has been shown to increase catecholamine release which may partially explain favor to anaerobic tasks (Yamamoto et al., 2003). This could be supported by the present meta-analysis results where pre-task music presented high practical effect on jumping performance even it was not statistically significant. Possibly, due to the short duration and volume of exercises performed after listening to music that fatigue was lower to alter performance when music effects dominate (Bigliassi, 2015). Alternatively, being efficient in explosive movements is based on an optimal psychological state which may be enhanced by listening to music even when it is performed prior to performance (Ballmann et al., 2021).

Regarding the importance of a positive psychological state during exercise, music was considered as an efficient strategy to increase sport activities commitment, even performed at high-intensities (Clark et al., 2015; Hutchinson et al., 2018) and may positively affect performance (Castaneda-Babarro et al., 2020). The present meta-analysis highlights fatigue symptom decreases and that affective valence scores were directed toward the positive end of the scale when pre-task music was used. The positive effect of music toward affective valence supported previous findings reporting moderate effect of music on FS (Terry et al., 2020). The use of simulative or motivational music implicates the brain stem reflex, stimulating the central nervous system in a manner reflecting physiological arousal increases which is associated with high-intensity activity (Karageorghis and Jones, 2014). Furthermore, when listening to music, the phenomenon of emotional contagion may occur in a manner that involves the exerciser or athlete to recognize the music’s emotional elements (Castaneda-Babarro et al., 2020). Bishop et al. (2013) indicated that listening to motivational music stimulates the primary auditory membrane parts and cerebellum responsible for processing emotions, managing movement or movement patterns control. Their mutual stimulation with music can be one of the main reasons for their effectiveness in listeners’ performance (Bishop et al., 2013).

Music has been shown likely to enhance motivation and arousal for sports (Karageorghis and Terry, 1997), which are the underlying psychological factors to maximize physical effort and performance (Anshel, 2019). In the present systematic review, evidence has consistently supported the idea that listening to music improves motivation to exercise (Smirmaul et al., 2014; Karow et al., 2020; Ballmann et al., 2021). Smirmaul et al. (2014) showed that trained swimmers who listened to music during warm-up felt more motivated prior to exercise and subsequently swam faster in the 200 m trial. From the other side, Karow et al. (2020) reported higher power output and speed in a rowing exercise related to higher motivation after listening to preferred music during warm-up. Such motivational effects of pre-task or warm-up music may not only be limited to pre- exercise, but this motivation may last throughout the exercise bout (Ballmann et al., 2021). Therefore, motivation enhancement reported by these previous studies likely led to increased effort and improved velocity and power output (Ballmann, 2021). As well, the ability to increase arousal and calm down was clearly associated with pre-task music (Bigliassi et al., 2015). Interestingly, previous meta-analysis showed the effectiveness of music to lower both physiological stress-related arousal (e.g., blood pressure, heart rate, and hormone levels) and psychological stress-related experiences (e.g., state anxiety, restlessness or nervousness) in various populations and settings (de Witte et al., 2020). Moreover, another meta-analysis showed that listening to music induced an overall significant large effect on alleviating anxiety (Harney et al., 2022). Such results had received consensus from pre-task music investigations where music reduced anxiety (Aloui et al., 2015), negative mood states (Khemila et al., 2021; Gavanda et al., 2022) and increased vigor (Chtourou et al., 2017). From a physiological point of view, pre-task music was associated with reduced post-exercise cortisol (Khemila et al., 2021) and maintained heart rate values (Jarraya et al., 2012; Arazi et al., 2015; Bigliassi et al., 2015; Jebabli et al., 2022, 2023) despite performance improvements. Decreased cortisol concentration may serve as an underlying mechanism for reducing fatigue (Ghaderi et al., 2015). Moreover, improved parasympathetic recovery (Heart rate variability analysis) was found to be the cause of music calming effect (Bigliassi et al., 2015).

Affective responses to exercise are jointly influenced by cognitive factors, such as physical self-efficacy and interoceptive (e.g., muscular or respiratory) cues that reach the affective centers of the brain via sub-cortical pathways (Ekkekakis, 2003). Using stimulating music, evidence shows improved cognitive performances during simple and complex tasks (Bishop et al., 2013; Jarraya and Jarraya, 2019; Khemila et al., 2021). The improvement of reaction time, attention allocation, and visual perception were the cognitive skills connected with pre-task music effects. From a neurological approach, music yielded persistent activation in the basal ganglia, which is implicated in effective decision-making and preparation for action in sport (Yarrow et al., 2009). In addition, the use of fast and loud music induces a brain state whereby an individual vigorously monitors his/her environment, and swiftly identifies relevant targets (Bishop et al., 2013).

Regarding RPE, the significant influence of music on perceived exertion was explained primarily by its ability to distract exercisers from unpleasant, fatigue-related sensations (Terry et al., 2020). However, using pre-task music stimulations, the current meta-analysis revealed a trivial non-significant effect on RPE. Although the benefits of music on RPE have been observed even during high-intensity activity (Bigliassi et al., 2012; Bentouati et al., 2022), its distraction effect can be denied by powerful interceptive signals of physical discomfort associated with the activity and the benefit to RPE may be lost (Stork et al., 2015). It is possible that, to maximize these benefits of shifting an external focus, music may need to be played simultaneously with, rather than before, the exercise task (Ballmann et al., 2021). This effect of the listening time could explain the significant effect of music on RPE when including data from during exercise (Terry et al., 2020). The effect of pre-task music has been reported to decrease over time due to fatigue’s increase (Aloui et al., 2015; Bigliassi, 2015). In fact, fatigue’s increase is generally stronger than the music effects, which means that it is only “a matter of time” until fatigue-related symptoms overcome the ergogenic effects of music (Rejeski, 1985; Bigliassi, 2015). Therefore, the luck of music analgesic effects could be supported by the intensity dependent theory (Bigliassi et al., 2012). Since effort intensity is often expressed as a percentage of maximal oxygen uptake, it seems that music reduces perceived exhaustion only up to 75% of maximal oxygen consumption, and these effects disappear quickly when intensity is above (Hutchinson and Karageorghis, 2013). Therefore, RPE values are likely maintained because self-pacing enables people to exercise at a determined RPE level throughout exercise (Fox et al., 2019).

### 4.2 Moderating variables

Situational and inter-individual variables are the mediators affecting the power of communication between a piece of music and the individual’s response (Hutchinson and Karageorghis, 2013). However, Terry et al. (2020) suggested that music is likely to induce positive responses during exercise on a fairly consistent basis regardless of personal, situational and musical characteristics. In the present meta-analysis, pre-task music effect on affective valence seems to be modulated by the selection process and the timing of variable measurement. The promotion of self-determined forms of behavioral regulation are likely to encourage the maintenance of physical activity behaviors (Ryan and Deci, 2017; Terry et al., 2020). Specifically, the purpose of selecting music is, generally speaking, to ensure that the individual objective is optimized (Eliakim et al., 2012). As a result, the music type that an individual can choose is of importance to determine the psycho-physiological impact of music on physical performance capacities (Işık et al., 2015). Jiang et al. (2016) showed that listening to self-selected music elicited stronger and more positive emotional responses regardless of the song’s valence (i.e., positive or negative) and arousal (i.e., high or low). This might be attributed to the fact that self-selected music was more preferred and familiar (Jiang et al., 2016). In fact, neural activation changes elicited by listening to music have been shown to be heavily dependent on the individual’s musical preference where greater emotional connection to preferred music may alter responses (Holler et al., 2012). In other words, listeners may respond differently to music melody and harmony based on their cultural background and familiarity level (Akhshabi and Rahimi, 2021). The higher performance under the preferred condition was also linked to expectations influence on the recorded effects (Ouergui et al., 2023b).

Individual factors such as sex (Nater et al., 2006; Karageorghis et al., 2018) and training status(Carlier et al., 2017) have been suggested to modulate the reaction to music during exercise. The present study revealed that music resulted in greater effects within males than females during explosive tasks. More potent effects of music in males could be related to their ability to develop higher muscular power while maintaining lower fatigue rate than females (Ouergui et al., 2023b). Regarding psychological responses, females may exhibit higher emotional sensitivity to musical stimuli compared to males (Nater et al., 2006). For instance, females fixate more attention to music’s rhythmic quality and movements than males, and males to the cultural bond of music more than their female counterparts (Karageorghis et al., 1999). Consequently, since the majority of examined studies used pre-selected music, male responses were positively altered maybe due to their higher objectivity (Nater et al., 2006). Considering training status, the influence of music on performance was reported to decrease significantly with increased fitness level (Eliakim et al., 2007). There is evidence that highly trained exercisers or elite athletes tend to associate rather than dissociate (Gabana et al., 2014) and then their RPE scores decrease due to music, even at high work intensities (Terry et al., 2020). In the present meta-analysis, the effects of pre-task music on physical performance outcomes (i.e., completion time, RPP and RMP) were greater on active than trained, whereas its effect on affective valence was greater in trained than active subjects. This result contradicts what was reported by Smirmaul (2017) where pre-task music effects were consistent regardless the fitness levels of individuals (i.e., varied from physically active students to national-level athletes). Our results could be attributed to the fact that trained subjects reached their top physical fitness level and then cannot benefit from music ergogenic effect (Berthelot et al., 2015). Thus, our findings could suggest that in highly trained subjects, pre-task music benefits on physical performances are highly mediated by its ergogenic potential on affective states (Carlier et al., 2017; Ballmann, 2021). This might be supported by the fact that music brought greater pleasure to the high than to the low tolerant participants (Carlier et al., 2017).

Regarding music intrinsic features, Terry et al. (2020) reported that fast-tempo music was associated with greater benefits than slow-tempo music. This is reasonable, since people express a preference for keeping the tempo relatively high during intense exercise (Thaut, 2013). Given the high-energy/activation state typically required for optimal performance in exercise or sport, the stronger effect of fast-tempo music reflects what we know about physiological arousal and musical aesthetics (Karageorghis, 2020). Yamamoto et al. (2003) showed that slow-rhythm music decreased excitement, while fast-rhythm music increased it. This rise in physiological excitement might be related to upbeat music ability to boost the heartbeat immediately before activity (Eliakim et al., 2007; Puad et al., 2020). Fast-rhythm music automatically stimulates the listener by activating the central nervous system, regardless of how the music is evaluated (Van Dyck, 2019). However, the present meta-analyses results did not support the moderating effects of music tempo as reported by Terry et al. (2020). It is important to note that when the power of physiological feedback signals overshadows the attention processes, the music ability to maintain physiological effects in strenuous physical activity is greatly altered (Ekkekakis, 2003). Since music is played before task execution, its tempo moderating effects may be dependent on listener’ affective memory and dissociation ability (Bishop et al., 2013; Stork et al., 2015).

### 4.3 Strengths and limitations

This is the first systematic review with meta-analysis addressing the acute effects of pre-task music on physical performance, cognitive aspects, and psycho-physiological responses associated to exercise and sport fields. The present investigation includes a comprehensive exposure of the available literature and a careful assessment of its importance as well as the presentation of the results from trials with moderate to high methodological quality. In addition, meta-analysis was conducted using a robust estimate model with the inclusion of all the available observations, which is the recommended approach to deal with dependency and show unbiased results (Assink and Wibbelink, 2016; Moeyaert et al., 2017). However, some limitations should be acknowledged in the present systematic review. In fact, due to the lack of sufficient information about music’ characteristics (e.g., intensity of music, type of music), it is impossible to make perfect subgroups comparisons. Moreover, a new aspect observed in this study is that these studies (Bishop et al., 2013; Smirmaul et al., 2014; Jarraya and Jarraya, 2019; Ballmann et al., 2021; Gavanda et al., 2022) used pre-task music at rest rather than during the warm-up, raising a topic for further discussion. Another limitation of the included studies was the lack of controlling/reporting methodological procedures in sufficient details. For instance, information regarding music’s characteristics such as intensity and the time between music exposure and task initiation was not sufficiently acknowledged. Regarding the mechanisms behind pre-task music effects, none of the included studies has directly measured dissociation, leaving its contribution to the music modulating effect on performance unclear at this time. Furthermore, despite motivation was the most common mechanism to explain warm-up music ergogenic potential, only three studies (Smirmaul et al., 2014; Karow et al., 2020; Ballmann et al., 2021) measured motivation level while the other ones take it as evident.

### 4.4 Future research perspectives

Pre-task music application remains restricted to exercise domain and less investigated in sport events (i.e., luck of investigations with competitions). This is why it would be appropriate to conduct further studies about the ergogenic effect of music on sport events. Moreover, even specificity is crucial training principle, it is clear that most of studies about pre-task music effects used generic rather than specific tests, which may limit generalization of results to specific conditions. Moreover, pre-task music exposure at rest vs. during the warm-up could generate different responses. In addition, determining the optimal time between pre-task music exposure and beginning of the task could be an interesting factor in music topics. Furthermore, as the objective in sports settings is to improve performances as much as possible, combining pre-task music with other ergogenic aid would be an attractive strategy.

## 5 Conclusion

As a psycho-active stimulus, warm-up music could be an effective strategy to enhance several aspects of performance (predominantly anaerobic tasks were the most affected), associated psychological responses and delay the fatigue-related symptoms. Such benefits of warm-up music could present implications on competition and training settings. Specifically, as a way to deal with psychological stress anticipated before competition, warm–up music could be highly attractive for elite athletes where minor differences determine the winner. Moreover, starting training sessions with high level of motivation and power help to achieve training objectives with lower cost, especially, for less trained subjects. However, pre-task music is a sensitive stimulus, which could be influenced by intrinsic and situational factors. Coaches seeking to improve their athletes’ performances and prepare them to cope with training and competitions demands using pre-task music are recommended to give them the opportunity to select their preferred music since subjects’ characteristics (e.g., sex, training status) seems to be the most moderating factors with varied preference level. In addition, since warm-up music effects depends on listeners’ sex, findings from one sample sex should not be generalized to the other one and taken with caution. Moreover, users are recommended to improve the quality of their music selection process by considering the music’s motivational properties and working with a guiding rationale. Even under preferred conditions, controlling the intrinsic components (e.g., volume, genre, melody) of the selected music could help to explain, in part, the varied responses across listeners. Furthermore, explaining warm-up music benefits should be articulated on measured responses (e.g., physiological, neurological, and psychological) that should be taken into consideration to explain the inter-individual variability and the existence of responders and non-responders to the stimulus.

## Data availability statement

The raw data supporting the conclusions of this article will be made available by the authors, without undue reservation.

## Author contributions

SD: Conceptualization, Data curation, Formal analysis, Investigation, Methodology, Resources, Software, Writing – original draft, Writing – review & editing. IO: Conceptualization, Investigation, Methodology, Resources, Validation, Writing – original draft, Writing – review & editing. CB: Investigation, Validation, Writing – original draft, Writing – review & editing. HM: Investigation, Writing – review & editing. KT: Investigation, Writing – review & editing. LPA: Investigation, Writing – review & editing. HC: Conceptualization, Investigation, Validation, Writing – original draft, Writing – review & editing.

## References

[ref1] Akhshabi M. Rahimi M. (2021). The impact of music on sports activities: a scoping review. J. N. Stud. Sport Manage. 2, 274–285. doi: 10.22103/JNSSM.2021.18566.1045

[ref2] Aloui A. Briki W. Baklouti H. Chtourou H. Driss T. Chaouachi A.. (2015). Listening to music during warming-up counteracts the negative effects of ramadan observance on short-term maximal performance. PLoS One 10:e0136400. doi: 10.1371/journal.pone.0136400, PMID: 26301508 PMC4547754

[ref3] Anshel M. H. (2019). “Cognitive and behavioral strategies to promote exercise performance” in APA Handbook of Sport and Exercise Psychology, Volume 2: Exercise Psychology. eds. Anshel M. H. Petruzzello S. J. Labbé E. E., vol. 2 (Washington, DC, USA: American Psychological Association), 667–689.

[ref4] Arazi H. Asadi A. Purabed M. (2015). Physiological and psychophysical responses to listening to music during warm-up and circuit-type resistance exercise in strength trained men. J. Sports Med. 2015, 1–6. doi: 10.1155/2015/389831 PMC4590888 26464896

[ref5] Ardern C. L. Buttner F. Andrade R. Weir A. Ashe M. C. Holden S.. (2022). Implementing the 27 PRISMA 2020 statement items for systematic reviews in the sport and exercise medicine, musculoskeletal rehabilitation and sports science fields: the PERSiST (implementing Prisma in exercise, rehabilitation, sport medicine and SporTs science) guidance. Br. J. Sports Med. 56, 175–195. doi: 10.1136/bjsports-2021-103987, PMID: 34625401 PMC8862073

[ref6] Assink M. Wibbelink C. J. (2016). Fitting three-level meta-analytic models in R: a step-by-step tutorial. Quant. Methods Psychol. 12, 154–174. doi: 10.20982/tqmp.12.3.p154

[ref7] Atkinson G. Wilson D. Eubank M. (2004). Effects of music on work-rate distribution during a cycling time trial. Int. J. Sports Med. 25, 611–615. doi: 10.1055/s-2004-815715 15532005

[ref8] Ballmann C. G. (2021). The influence of music preference on exercise responses and performance: a review. J Funct Morphol Kinesiol 6:33. doi: 10.3390/jfmk6020033, PMID: 33917781 PMC8167645

[ref9] Ballmann C. G. Favre M. L. Phillips M. T. Rogers R. R. Pederson J. A. Williams T. D. (2021). Effect of pre-exercise music on bench press power, velocity, and repetition volume. Percept. Mot. Skills 128, 1183–1196. doi: 10.1177/00315125211002406, PMID: 33722102

[ref10] Ballmann C. G. Maynard D. J. Lafoon Z. N. Marshall M. R. Williams T. D. Rogers R. R. (2019). Effects of listening to preferred versus non-preferred music on repeated Wingate anaerobic test performance. Sports 7:185. doi: 10.3390/sports7080185, PMID: 31362419 PMC6723041

[ref11] Bayrakdaroğlu S. Eken Ö. Yagin F. H. Bayer R. Gulu M. Akyildiz Z.. (2022). Warm up with music and visual feedback can effect Wingate performance in futsal players. BMC Sports Sci. Med. Rehabil. 14:205. doi: 10.1186/s13102-022-00601-3, PMID: 36474255 PMC9727888

[ref12] Belkhir Y. Rekik G. Chtourou H. Souissi N. (2019). Listening to neutral or self-selected motivational music during warm-up to improve short-term maximal performance in soccer players: effect of time of day. Physiol. Behav. 204, 168–173. doi: 10.1016/j.physbeh.2019.02.033 30817975

[ref13] Belkhir Y. Rekik G. Chtourou H. Souissi N. (2020). Effect of listening to synchronous versus motivational music during warm-up on the diurnal variation of short-term maximal performance and subjective experiences. Chronobiol. Int. 37, 1611–1620. doi: 10.1080/07420528.2020.1797764 32741226

[ref14] Belkhir Y. Rekik G. Chtourou H. Souissi N. (2021). Does warming up with different music tempos affect physical and psychological responses? The evidence from a chronobiological study. J. Sports Med. Phys. Fitness 62, 149–156. doi: 10.23736/S0022-4707.21.12093-6, PMID: 33555672

[ref15] Bentouati E. Romdhani M. Khemila S. Chtourou H. Souissi N. (2022). The effects of listening to non-preferred or self-selected music during short-term maximal exercise at varied times of day. Percept. Mot. Skills 130, 539–554. doi: 10.1177/00315125221142662 36458504

[ref16] Berthelot G. Sedeaud A. Marck A. Antero-Jacquemin J. Schipman J. Sauliere G.. (2015). Has athletic performance reached its peak? Sports Med. 45, 1263–1271. doi: 10.1007/s40279-015-0347-2, PMID: 26094000 PMC4536275

[ref17] Bigliassi M. (2015). Use the brain: complementary methods to analyse the effects of motivational music. Front. Hum. Neurosci. 9:508. doi: 10.3389/fnhum.2015.00508, PMID: 26441605 PMC4584946

[ref18] Bigliassi M. Dantas J. Carneiro J. Smirmaul B. Altimari L. (2012). Influence of music and its moments of application on performance and psychophysiological parameters during a 5 km time trial. Rev. Andal. Med. Deport. 5, 83–90. doi: 10.1016/S1888-7546(12)70013-8

[ref19] Bigliassi M. Leon-Dominguez U. Buzzachera C. F. Barreto-Silva V. Altimari L. R. (2015). How does music aid 5 km of running? J Strength 29, 305–314. doi: 10.1519/JSC.0000000000000627 25029009

[ref20] Bishop D. T. Wright M. J. Karageorghis C. I. (2013). Tempo and intensity of pre-task music modulate neural activity during reactive task performance. Psychol. Music 42, 714–727. doi: 10.1177/0305735613490595

[ref21] Carlier M. Delevoye-Turrell Y. On Behalf of the Fun2move Consortium (2017). Tolerance to exercise intensity modulates pleasure when exercising in music: the upsides of acoustic energy for high tolerant individuals. PLoS One 12:e0170383. doi: 10.1371/journal.pone.0170383, PMID: 28248980 PMC5331955

[ref22] Castaneda-Babarro A. Marques-Jimenez D. Calleja-Gonzalez J. Viribay A. Leon-Guereno P. Mielgo-Ayuso J. (2020). Effect of listening to music on Wingate anaerobic test performance. A systematic review and Meta-analysis. Int. J. Environ. Res. Public Health 17:4564. doi: 10.3390/ijerph17124564, PMID: 32599941 PMC7344562

[ref23] Cheung M. W. (2014). Modeling dependent effect sizes with three-level meta-analyses: a structural equation modeling approach. Psychol. Methods 19, 211–229. doi: 10.1037/a0032968, PMID: 23834422

[ref24] Chtourou H. Chaouachi A. Hammouda O. Chamari K. Souissi N. (2012a). Listening to music affects diurnal variation in muscle power output. Int. J. Sports Med. 33, 43–47. doi: 10.1055/s-0031-1284398 22134883

[ref25] Chtourou H. Hmida C. Souissi N. (2017). Effect of music on short-term maximal performance: sprinters vs. long distance runners. Sport Sci. Health 13, 213–216. doi: 10.1007/s11332-017-0357-6

[ref26] Chtourou H. Jarraya M. Aloui A. Hammouda O. Souissi N. (2012b). The effects of music during warm-up on anaerobic performances of young sprinters. Sci. Sports 27, e85–e88. doi: 10.1016/j.scispo.2012.02.006

[ref27] Clark I. N. Baker F. A. Taylor N. F. (2015). The modulating effects of music listening on health-related exercise and physical activity in adults: a systematic review and narrative synthesis. Nord. J. Music. Ther. 25, 76–104. doi: 10.1080/08098131.2015.1008558

[ref28] Cook R. D. (1977). Detection of influential observation in linear regression. Technometrics 19, 15–18.

[ref29] de Morton N. A. (2009). The PEDro scale is a valid measure of the methodological quality of clinical trials: a demographic study. Aust. J. Physiother. 55, 129–133. doi: 10.1016/S0004-9514(09)70043-1 19463084

[ref30] de Witte M. Spruit A. van Hooren S. Moonen X. Stams G. J. (2020). Effects of music interventions on stress-related outcomes: a systematic review and two meta-analyses. Health Psychol. Rev. 14, 294–324. doi: 10.1080/17437199.2019.1627897 31167611

[ref31] Drevon D. Fursa S. R. Malcolm A. L. (2017). Intercoder reliability and validity of WebPlotDigitizer in extracting graphed data. Behav. Modif. 41, 323–339. doi: 10.1177/0145445516673998, PMID: 27760807

[ref32] Ekkekakis P. (2003). Pleasure and displeasure from the body: perspectives from exercise. Cogn Emot 17, 213–239. doi: 10.1080/02699930302292, PMID: 29715726

[ref33] Eliakim M. Meckel Y. Gotlieb R. Nemet D. Eliakim A. (2012). Motivational music and repeated sprint ability in junior basketball players. Acta Kinesiologiae Univ. Tartuensis 18, 29–38. doi: 10.12697/akut.2012.18.04

[ref34] Eliakim M. Meckel Y. Nemet D. Eliakim A. (2007). The effect of music during warm-up on consecutive anaerobic performance in elite adolescent volleyball players. Int. J. Sports Med. 28, 321–325. doi: 10.1055/s-2006-924360, PMID: 17024625

[ref35] Fernández-Castilla B. Declercq L. Jamshidi L. Beretvas S. N. Onghena P. Van den Noortgate W. (2021). Detecting selection Bias in Meta-analyses with multiple outcomes: a simulation study. J. Exp. Educ. 89, 125–144. doi: 10.1080/00220973.2019.1582470 32808180

[ref36] Fox R. P. Michael T. J. Weideman C. A. Hanson N. J. (2019). Effect of listening to music during a warmup on anaerobic test performance. Sport Sci. Health 15, 369–373. doi: 10.1007/s11332-019-00525-5

[ref37] Gabana N. T. Van Raalte J. L. Hutchinson J. C. Brewer B. W. Petitpas A. J. (2014). The effects of music and a coxswain on attentional focus, perceived exertion, motivation, and performance during a 1,000 m ergometer rowing Sprint. J. Appl. Sport Psychol. 27, 288–300. doi: 10.1080/10413200.2014.993775

[ref38] Garbisu-Hualde A. Santos-Concejero J. (2021). Post-activation potentiation in strength training: a systematic review of the scientific literature. J. Hum. Kinet. 78, 141–150. doi: 10.2478/hukin-2021-0034, PMID: 34025872 PMC8120977

[ref39] Gavanda S. Hosang T. Wagener S. Sönmez N. Kayser I. Knicker A. (2022). The influence of relaxing and self-selected stimulating music on vertical jump performance in male volleyball players. Int. J. Exerc. Sci. 15, 15–24. 36895325 10.70252/PFNC1124 PMC9987433

[ref40] Ghaderi M. Nikbakht H. Chtourou H. Jafari M. Chamari K. (2015). Listening to motivational music: lactate and cortisol response to a single circuit resistance exercise for young male athletes. S. Afr. J. Res. Sport Phys. Educ. Recreation 37, 33–45.

[ref41] Ghazel N. Souissi A. Chtourou H. Aloui G. Souissi N. (2022). The effect of music on short-term exercise performance during the different menstrual cycle phases in female handball players. Res. Sports Med. 30, 50–60. doi: 10.1080/15438627.2020.1860045 33291988

[ref42] Gordon C. L. Cobb P. R. Balasubramaniam R. (2018). Recruitment of the motor system during music listening: an ALE meta-analysis of fMRI data. PLoS One 13:e0207213. doi: 10.1371/journal.pone.0207213, PMID: 30452442 PMC6242316

[ref43] Gucciardi D. F. Lines R. L. J. Ntoumanis N. (2021). Handling effect size dependency in meta-analysis. Int. Rev. Sport Exerc. Psychol. 15, 152–178. doi: 10.1080/1750984x.2021.1946835

[ref44] Harney C. Johnson J. Bailes F. Havelka J. (2022). Is music listening an effective intervention for reducing anxiety? A systematic review and meta-analysis of controlled studies. Music. Sci. 27, 278–298. doi: 10.1177/10298649211046979

[ref45] Hedges L. V. Olkin I. (2014). Statistical Methods for Meta-Analysis. Cambridge, MC, USA: Academic Press.

[ref46] Higgins J. P. Thomas J. Chandler J. Cumpston M. Li T. Page M. J.. (2019). Cochrane Handbook for Systematic Reviews of Interventions. Hoboken, NJ, USA: John Wiley & Sons, Inc. 10.1002/14651858.ED000142 PMC10284251 31643080

[ref47] Higgins J. P. T. Thompson S. G. Deeks J. J. Altman D. G. (2003). Measuring inconsistency in meta-analyses. BMJ 327, 557–560. doi: 10.1136/bmj.327.7414.557, PMID: 12958120 PMC192859

[ref48] Holler Y. Thomschewski A. Schmid E. V. Holler P. Crone J. S. Trinka E. (2012). Individual brain-frequency responses to self-selected music. Int. J. Psychophysiol. 86, 206–213. doi: 10.1016/j.ijpsycho.2012.09.005, PMID: 23000014

[ref49] Hopkins W. G. Hawley J. A. Burke L. M. (1999). Design and analysis of research on sport performance enhancement. Med. Sci. Sports Exerc. 31, 472–485. doi: 10.1097/00005768-199903000-00018 10188754

[ref50] Hutchinson J. C. Jones L. Vitti S. N. Moore A. Dalton P. C. O'Neil B. J. (2018). The influence of self-selected music on affect-regulated exercise intensity and remembered pleasure during treadmill running. Sport Exerc. Perform. Psychol. 7, 80–92. doi: 10.1037/spy0000115

[ref51] Hutchinson J. C. Karageorghis C. I. (2013). Moderating influence of dominant attentional style and exercise intensity on responses to asynchronous music. J. Sport Exerc. Psychol. 35, 625–643. doi: 10.1123/jsep.35.6.625, PMID: 24334323

[ref52] IntHout J. Ioannidis J. P. A. Rovers M. M. Goeman J. J. (2016). Plea for routinely presenting prediction intervals in meta-analysis. BMJ Open 6:e010247. doi: 10.1136/bmjopen-2015-010247, PMID: 27406637 PMC4947751

[ref53] Işık Ö. Ersöz Y. Pazan M. Ocak Y. (2015). The effect of motivational music on Wingate anaerobic test performance. J. Hum. Sci. 12, 513–520. doi: 10.14687/ijhs.v12i2.3254

[ref54] Jarraya M. Chtourou H. Aloui A. Hammouda O. Chamari K. Chaouachi A.. (2012). The effects of music on high-intensity short-term exercise in well trained athletes. Asian J. Sports Med. 3, 233–238. doi: 10.5812/asjsm.34543, PMID: 23342221 PMC3525819

[ref55] Jarraya S. Jarraya M. (2019). The effects of music and the time-of-day on cognitive abilities of tennis player. Int. J. Sport Exe. Psychol. 17, 185–196. doi: 10.1080/1612197X.2017.1292299

[ref56] Jebabli N. Ben Aabderrahman A. Boullosa D. Chtourou H. Ouerghi N. Rhibi F.. (2023). Listening to music during a repeated sprint test improves performance and psychophysiological responses in healthy and physically active male adults. BMC Sports Sci. Med. Rehabil. 15:21. doi: 10.1186/s13102-023-00619-1, PMID: 36810282 PMC9945598

[ref57] Jebabli N. Zouhal H. Boullosa D. Govindasamy K. Tourny C. Hackney A. C.. (2022). The effects of preferred music and its timing on performance, pacing, and psychophysiological responses during the 6-min test. J. Hum. Kinet. 82, 123–133. doi: 10.2478/hukin-2022-0038 36196352 PMC9465734

[ref58] Jiang J. Rickson D. Jiang C. (2016). The mechanism of music for reducing psychological stress: music preference as a mediator. Arts Psychother. 48, 62–68. doi: 10.1016/j.aip.2016.02.002

[ref59] Ka-Lok I. Chen Y.-S. Lu W.-A. Bezerra P. (2020). Acute effects of self-selected music intervention on recovery of autonomic functions and anxiety after submaximal intensity of short-term cycling. Int. Multidisciplinary J. CREA 1, 51–63. doi: 10.35869/ijmc.v1i1.2848

[ref60] Karageorghis C. I. (2020). “Music-related interventions in the exercise domain: a theory-based approach” in Handbook of Sport Psychology. eds. Tenenbaum G. Eklund R. C. (Hoboken, NJ, USA: John Wiley& Sons, Inc.), 929–949.

[ref61] Karageorghis C. I. Bigliassi M. Tayara K. Priest D.-L. Bird J. M. (2018). A grounded theory of music use in the psychological preparation of academy soccer players. Sport Exerc. Perform. Psychol. 7, 109–127. doi: 10.1037/spy0000110

[ref62] Karageorghis C. I. Jones L. (2014). On the stability and relevance of the exercise heart rate–music-tempo preference relationship. Psychol. Sport Exerc. 15, 299–310. doi: 10.1016/j.psychsport.2013.08.004 38608851

[ref63] Karageorghis C. I. Kuan G. Schiphof-Godart L. (2021). “Music in sport: from conceptual underpinnings to applications” in Essentials of Exercise and Sport Psychology: An Open Access Textbook. eds. Zenko Z. Jones L. (Society for Transparency, Openness, and Replication in Kinesiology), 530–564.

[ref64] Karageorghis C. I. Priest D. L. (2012). Music in the exercise domain: a review and synthesis (part I). Int. Rev. Sport Exerc. Psychol. 5, 44–66. doi: 10.1080/1750984X.2011.631026, PMID: 22577472 PMC3339578

[ref65] Karageorghis C. I. Terry P. C. (1997). The psychophysical effects of music in sport and exercise: a review. J. Sport Behav. 20:54.

[ref66] Karageorghis C.I. Terry P.C. (2011). Inside Sport Psychology. Human Kinetics Champaign, IL.

[ref67] Karageorghis C. I. Terry P. C. Lane A. M. (1999). Development and initial validation of an instrument to assess the motivational qualities of music in exercise and sport: the Brunel music rating inventory. J. Sports Sci. 17, 713–724. doi: 10.1080/026404199365579, PMID: 10521002

[ref68] Karow M. C. Rogers R. R. Pederson J. A. Williams T. D. Marshall M. R. Ballmann C. G. (2020). Effects of preferred and nonpreferred warm-up music on exercise performance. Percept. Mot. Skills 127, 912–924. doi: 10.1177/0031512520928244, PMID: 32493179

[ref69] Khemila S. Abedelmalek S. Romdhani M. Souissi A. Chtourou H. Souissi N. (2021). Listening to motivational music during warming-up attenuates the negative effects of partial sleep deprivation on cognitive and short-term maximal performance: effect of time of day. Chronobiol. Int. 38, 1052–1063. doi: 10.1080/07420528.2021.1904971, PMID: 33874838

[ref70] Loizou G. Karageorghis C. I. (2015). Effects of psychological priming, video, and music on anaerobic exercise performance. Scand. J. Med. Sci. Sports 25, 909–920. doi: 10.1111/sms.12391 25556962

[ref71] Maher C. G. Sherrington C. Herbert R. D. Moseley A. M. Elkins M. (2003). Reliability of the PEDro scale for rating quality of randomized controlled trials. Phys. Ther. 83, 713–721. doi: 10.1093/ptj/83.8.713, PMID: 12882612

[ref72] Moeyaert M. Ugille M. Natasha Beretvas S. Ferron J. Bunuan R. Van den Noortgate W. (2017). Methods for dealing with multiple outcomes in meta-analysis: a comparison between averaging effect sizes, robust variance estimation and multilevel meta-analysis. Int. J. Soc. Res. Methodol. 20, 559–572. doi: 10.1080/13645579.2016.1252189

[ref73] Nater U. M. Abbruzzese E. Krebs M. Ehlert U. (2006). Sex differences in emotional and psychophysiological responses to musical stimuli. Int. J. Psychophysiol. 62, 300–308. doi: 10.1016/j.ijpsycho.2006.05.011, PMID: 16828911

[ref74] Ouergui I. Jebabli E. Delleli S. Messaoudi H. Bridge C. A. Chtourou H.. (2023b). Listening to preferred and loud music enhances taekwondo physical performances in adolescent athletes. Percept. Mot. Skills 130, 1644–1662. doi: 10.1177/00315125231178067 37222224

[ref75] Ouergui I. Jebabli A. Messaoudi H. Delleli S. Chtourou H. Bouassida A.. (2023a). The effects of tempo and loudness variations during warm-up with music on perceived exertion, physical enjoyment and specific performances in male and female taekwondo athletes. PLoS One 18:e0284720. doi: 10.1371/journal.pone.0284720, PMID: 37104494 PMC10138780

[ref76] Page M. J. McKenzie J. E. Bossuyt P. M. Boutron I. Hoffmann T. C. Mulrow C. D.. (2021). The PRISMA 2020 statement: an updated guideline for reporting systematic reviews. J. Clin. Epidemiol. 134, 178–189. doi: 10.1016/j.jclinepi.2021.03.001 33789819

[ref77] Puad S. M. S. M. Ismail M. Nor M. A. M. Tumijan W. Kassim A. F. M. Amiruddin M. (2020). The effects of music during warm-up on anaerobic performance of football players. Int J Hum Mov 8, 477–482. doi: 10.13189/saj.2020.080621

[ref78] Pustejovsky J. E. Tipton E. (2022). Meta-analysis with robust variance estimation: expanding the range of working models. Prev. Sci. 23, 425–438. doi: 10.1007/s11121-021-01246-3, PMID: 33961175

[ref79] Rejeski W. J. (1985). Perceived exertion: an active or passive process? J. Sport Exerc. Psychol. 7, 371–378. doi: 10.1123/jsp.7.4.371

[ref80] Ryan R. M. Deci E. L. (2017). Self-Determination Theory: Basic Psychological Needs in Motivation, Development, and Wellness. New York City, NY, USA: Guilford Publications.

[ref81] Smirmaul B. P. (2017). Effect of pre-task music on sports or exercise performance. J. Sports Med. Phys. Fitness 57, 976–984. doi: 10.23736/s0022-4707.16.06411-2 27244132

[ref82] Smirmaul B. Dos Santos R. Da Silva Neto L. (2014). Pre-task music improves swimming performance. J. Sports Med. Phys. Fitness 55, 1445–1451. PMID: 25303170

[ref83] Spruit A. Assink M. van Vugt E. van der Put C. Stams G. J. (2016). The effects of physical activity interventions on psychosocial outcomes in adolescents: a meta-analytic review. Clin. Psychol. Rev. 45, 56–71. doi: 10.1016/j.cpr.2016.03.006, PMID: 27064552

[ref84] Stork M. J. Kwan M. Y. Gibala M. J. Martin Ginis K. A. (2015). Music enhances performance and perceived enjoyment of sprint interval exercise. Med. Sci. Sports Exerc. 47, 1052–1060. doi: 10.1249/MSS.0000000000000494, PMID: 25202850

[ref85] Terry P. C. Karageorghis C. I. Curran M. L. Martin O. V. Parsons-Smith R. L. (2020). Effects of music in exercise and sport: a meta-analytic review. Psychol. Bull. 146, 91–117. doi: 10.1037/bul0000216, PMID: 31804098

[ref86] Thaut M. (2013). Rhythm, Music, and the Brain: Scientific Foundations and Clinical Applications. Abingdon-on-Thames, UK: Taylor & Francis.

[ref87] Tounsi M. Jaafar H. Aloui A. Tabka Z. Trabelsi Y. (2019). Effect of listening to music on repeated-sprint performance and affective load in young male and female soccer players. Sport Sci. Health 15, 337–342. doi: 10.1007/s11332-018-0518-2

[ref88] Van Dyck E. (2019). Musical intensity applied in the sports and exercise domain: an effective strategy to boost performance? Front. Psychol. 10:1145. doi: 10.3389/fpsyg.2019.01145, PMID: 31156525 PMC6529527

[ref89] Viechtbauer W. (2010). Conducting meta-analyses in R with the metafor package. J. Stat. Softw. 36, 1–48. doi: 10.18637/jss.v036.i03

[ref90] Viechtbauer W. Cheung M. W. (2010). Outlier and influence diagnostics for meta-analysis. Res. Synth. Methods 1, 112–125. doi: 10.1002/jrsm.11 26061377

[ref91] Yamamoto T. Ohkuwa T. Itoh H. Kitoh M. Terasawa J. Tsuda T.. (2003). Effects of pre-exercise listening to slow and fast rhythm music on supramaximal cycle performance and selected metabolic variables. Arch. Physiol. Biochem. 111, 211–214. doi: 10.1076/apab.111.3.211.23464, PMID: 14972741

[ref92] Yarrow K. Brown P. Krakauer J. W. (2009). Inside the brain of an elite athlete: the neural processes that support high achievement in sports. Nat. Rev. Neurosci. 10, 585–596. doi: 10.1038/nrn2672 19571792

