# Supplementary material for: The effects of pre-task music on exercise performance and associated psycho-physiological responses: a systematic review with multilevel meta-analysis of controlled studies
Source: Front Psychol. 2023 Nov 23;14:1293783. doi: 10.3389/fpsyg.2023.1293783 (PMC10701429; doi:10.3389/fpsyg.2023.1293783)
Supplement: Supplementary file 2 [file Table_2.DOCX]

Supplementary Material

# Supplementary Figures and Tables

## Supplementary Figures


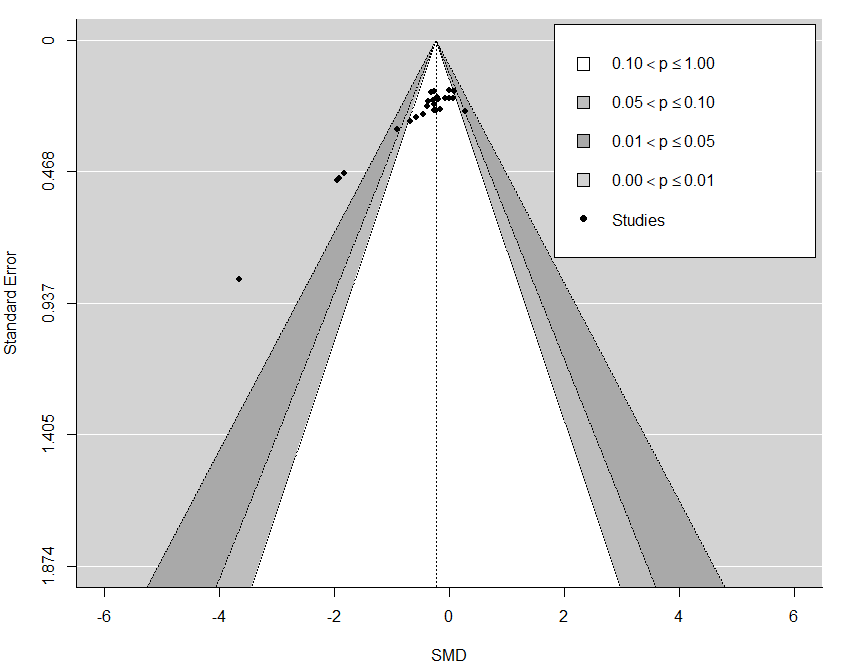


**Supplementary figure 1.** Funnel plot of pre-task music on completion time


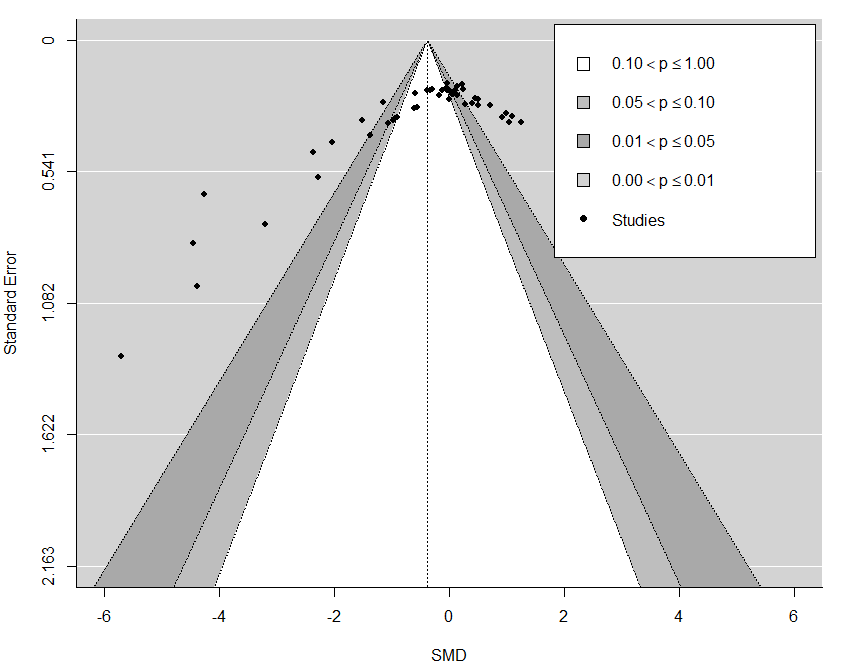


**Supplementary figure 2.** Funnel plot of pre-task music on performance decrement index


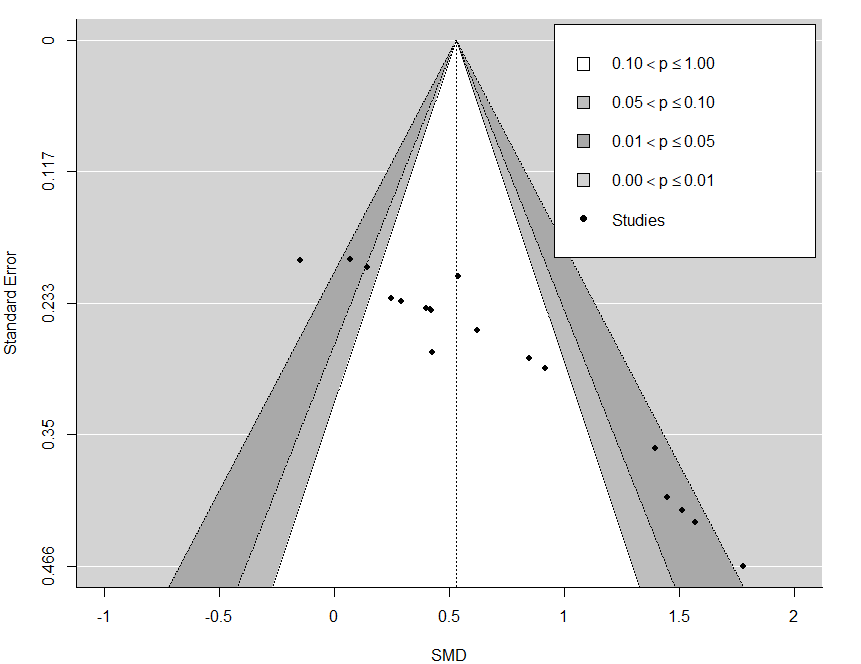


**Supplementary figure 3.** Funnel plot of pre-task music on relative peak power


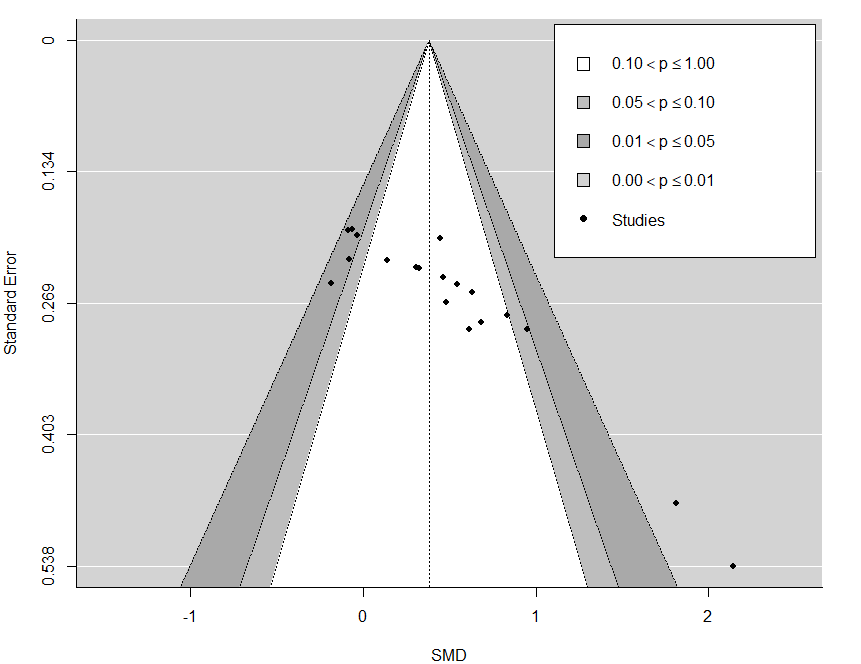


**Supplementary figure 4.** Funnel plot of pre-task music on relative mean power


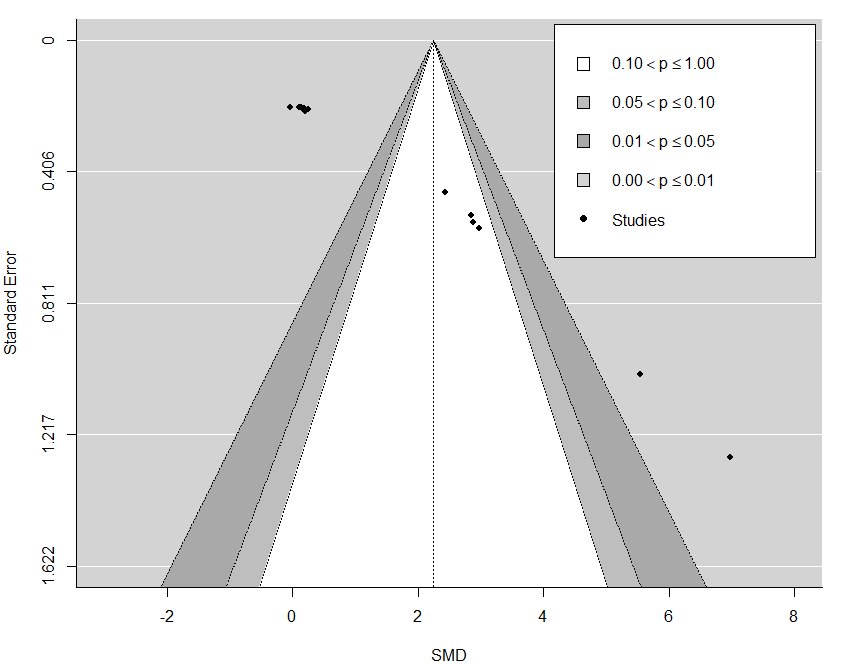


**Supplementary figure 5.** Funnel plot of pre-task music on jump height


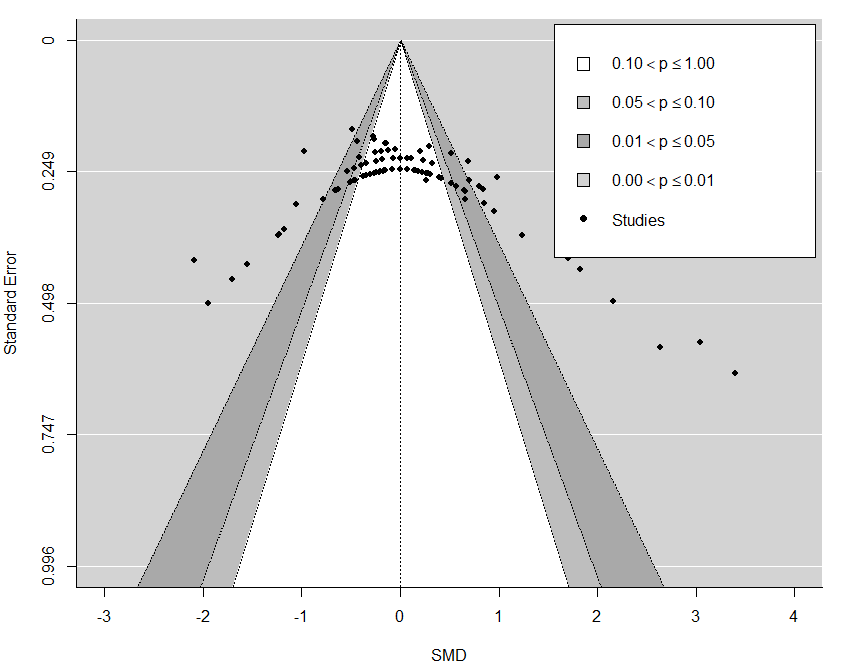


**Supplementary figure 6.** Funnel plot of pre-task music on RPE


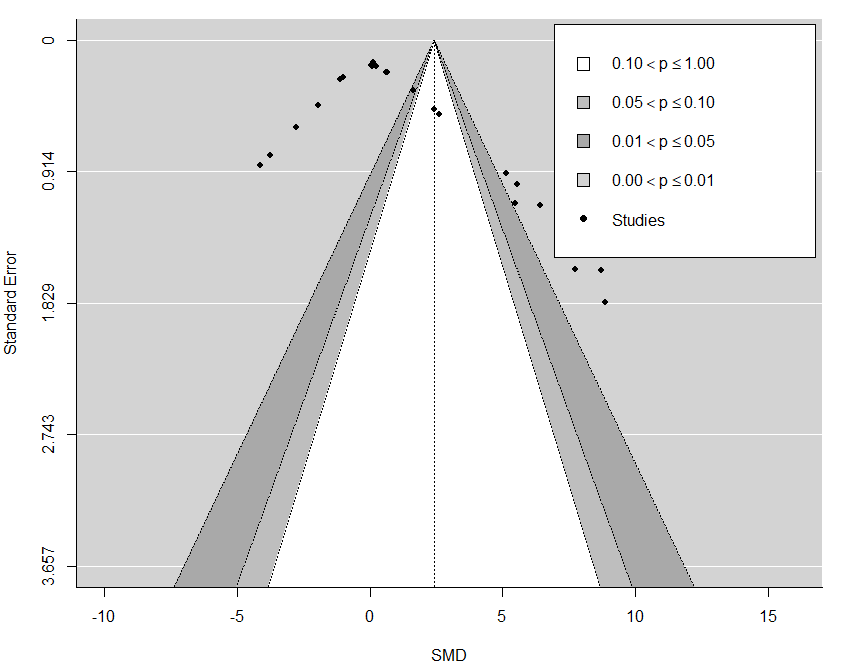


**Supplementary figure7.** Funnel plot of pre-task music on feeling scale


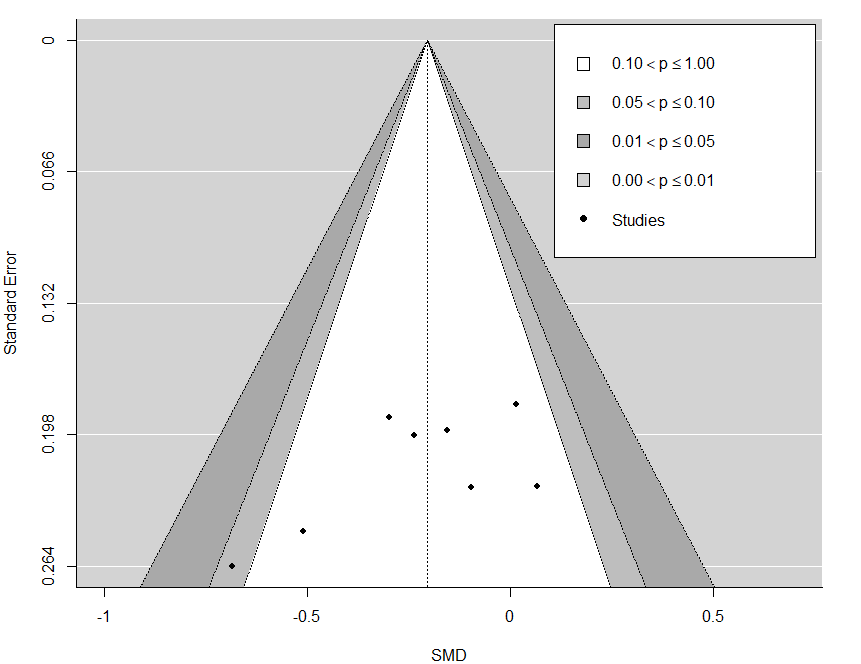


**Supplementary figure 8.** Funnel plot of pre-task music on fatigue symptoms

# Supplementary Tables

**Supplementary table 1.** Terms combinations and results on each database.

| **Database** | **Combinations** | **Results** |
| --- | --- | --- |
| Pubmed | ("warm up"[All Fields] OR "warm-up"[All Fields] OR "pre task"[All Fields] OR "pre-task"[All Fields] OR "pre exercise"[All Fields] OR "pre-exercise" [All Fields]) AND ("music"[MeSH Terms] OR "music"[All Fields]) AND ("exercise" [MeSH Terms] OR "exercise" [All Fields] OR "performance"[All Fields] OR "sports" [MeSH Terms] OR "sports"[All Fields]) | 68 |
| Cochrane library | ("warm-up" OR "warm up" OR "pre-task" OR "pre task" OR "pre-exercise" OR "pre exercise") AND (music) AND (exercise OR sports OR performance) | 96 |
| Scopus | ("warm-up" OR "warm up" OR "pre-task" OR "pre task" OR "pre-exercise" OR "pre exercise") AND (music) AND (exercise OR sports OR performance) | 119 |
| Web of Science | ("warm-up" OR "warm up" OR "pre-task" OR "pre task" OR "pre-exercise" OR "pre exercise") AND (music) AND (exercise OR sports OR performance) | 96 |
| Sport Discuss | ("warm-up" OR "warm up" OR "pre-task" OR "pre task" OR "pre-exercise" OR "pre exercise") AND (music) AND (exercise OR sports OR performance) | 87 |

**Supplementary table 2.** Full texts Excluded with reasons

| **Exclusion Reason** | **Studies excluded** |
| --- | --- |
| Uncontrolled studies | (Yamamoto et al., 2003; Ballmann et al., 2020; Meglic et al., 2021) |
| Longitudinal studies | (Pain et al., 2011; Hallett and Lamont, 2019) |
| Combined effects | (Rebadomia et al., 2019) |
| Conference paper | (Shojaei and Sangsari, 2010) |
| Virtual performance | (Wang et al., 2020; Wang et al., 2022) |
| Timing of intervention | (Bigliassi et al., 2017) |
| Subjective measurement | (Yeats et al., 2014) |

**Full texts references**

Ballmann, C.G., Cook, G.D., Hester, Z.T., Kopec, T.J., Williams, T.D., and Rogers, R.R. (2020). Effects of Preferred and Non-Preferred Warm-Up Music on Resistance Exercise Performance. *J Funct Morphol Kinesiol* 6(1). doi: 10.3390/jfmk6010003.

Bigliassi, M., Karageorghis, C.I., Wright, M.J., Orgs, G., and Nowicky, A.V. (2017). Effects of auditory stimuli on electrical activity in the brain during cycle ergometry. *Physiol Behav* 177**,** 135-147. doi: 10.1016/j.physbeh.2017.04.023.

Hallett, R., and Lamont, A. (2019). Evaluation of a motivational pre-exercise music intervention. *J Health Psychol* 24(3)**,** 309-320. doi: 10.1177/1359105316674267.

Meglic, C.E., Orman, C.M., Rogers, R.R., Williams, T.D., and Ballmann, C.G. (2021). Influence of Warm-Up Music Preference on Anaerobic Exercise Performance in Division I NCAA Female Athletes. *J Funct Morphol Kinesiol* 6(3). doi: 10.3390/jfmk6030064.

Pain, M.A., Harwood, C., and Anderson, R. (2011). Pre-Competition Imagery and Music: The Impact on Flow and Performance in Competitive Soccer. *The Sport Psychologist* 25(2)**,** 212-232. doi: 10.1123/tsp.25.2.212.

Rebadomia, F.M.L., Amparo, J.S.M.G., Reyes, J.P., Cobar, A.G.C., and Camarador, R.A. (2019). Effect of music with brainwave synchronizer on the performance of collegiate throwing athletes. *Sport Mont* 17(2)**,** 17-22. doi: 10.26773/smj.190603.

Shojaei, M., and Sangsari, M.M. (2010). Effect of listening to slow and fast rhythm music, during warm up on arousal and performance in elite basketball players. *Journal of Science & Medicine in Sport* 12**,** e112-e112.

Wang, H.T., Chen, Y.S., Rekik, G., Yang, C.C., Lai, M.S., and Tai, H.L. (2022). The effect of listening to preferred music after a stressful task on performance and psychophysiological responses in collegiate golfers. *PeerJ* 10**,** e13557. doi: 10.7717/peerj.13557.

Wang, H.T., Tai, H.L., Yang, C.C., and Chen, Y.S. (2020). Acute Effects of Self-Selected Music Intervention on Golf Performance and Anxiety Level in Collegiate Golfers: A Crossover Study. *Int J Environ Res Public Health* 17(20). doi: 10.3390/ijerph17207478.

Yamamoto, T., Ohkuwa, T., Itoh, H., Kitoh, M., Terasawa, J., Tsuda, T., et al. (2003). Effects of pre-exercise listening to slow and fast rhythm music on supramaximal cycle performance and selected metabolic variables. *Arch Physiol Biochem* 111(3)**,** 211-214. doi: 10.1076/apab.111.3.211.23464.

Yeats, J.T., Rhoads, M.C., Smith, M.A., and White, L.O. (2014). High School Volleyball Athletes' Perceptions of Creating and Using Pre-Competition Warm-Up Music. *Sport Science Review* 23(3/4)**,** 127-149.

**Supplementary table 3.** Moderators analysis for competion time

| **Moderator** | **group** | **SMD [95%CI]** | **F (df1, df2)** | **p** |
| --- | --- | --- | --- | --- |
| Selection |  |  | F(3, 1.22) = 1.16 | P=0.55 |
|  | SSM | -0.20 [-0.81, 0.41] |  |  |
|  | PSM | -0.23 [-0.91, 0.45] |  |  |
|  | Pseudo | -0.25 [-0.82, 0.32] |  |  |
| Time of day |  |  | F( 2, 3.23) = 2.49 | P=0.22 |
|  | Evening | -0.41 [-1.27, 0.45] |  |  |
|  | Unspecified | -0.12 [-0.34, 0.09] |  |  |
| Sex |  |  | F(3, 1.48) = 0.92 | P=0.58 |
|  | Male | -0.30 [-0.65, 0.05] |  |  |
|  | Female | -0.25 [-0.59, 0.09] |  |  |
|  | Mixed/Unspecified | -0.07 [-3.83, 3.69] |  |  |
| Test duration |  |  | F(2, 3.44) = 4.24 | P=0.12 |
|  | Short | -0.39 [-0.84, 0.05] |  |  |
|  | Longue | -0.13 [-0.32, 0.05] |  |  |
| Training status |  |  | F(2,1.56)=121.23 | p=0.02 |
|  | active | -0.30 [-0.50, -0.11] |  |  |
|  | trained | -0.21 [-0.53, 0.10] |  |  |
| Age |  | 0.06 [-0.02, 0.14] | F(1, 4.57) = 3.96 | P=0.11 |
| Exposure duration |  | -0.005 [-0.13, 0.12] | F(1, 2.04) = 0.03 | P=0.87 |

**Supplementary table 4.** Moderators analysis for performance decrement index

| Moderator | Group | SMD [95%CI] | F (df1, df2) | p |
| --- | --- | --- | --- | --- |
| Selection |  |  | F(3, 1.78) = 23.44 | P=0.05 |
|  | SSM | -1.35 [-2.94, 0.24] |  |  |
|  | PSM | -0.34 [-0.93, 0.24] |  |  |
|  | Pseudo | 0.50 [-1.37, 2.37] |  |  |
| Tempo |  |  | F(2, 3.37) = 1.25 | P=0.33 |
|  | Fast | -0.37 [-0.95, 0.20] |  |  |
|  | Slow | -0.52 [-1.70, 0.65] |  |  |
| Time of day |  |  | F(3, 7.17) = 0.54 | P=0.67 |
|  | Morning | -0.79 [-2.79, 1.21] |  |  |
|  | Evening | -0.27 [-0.90, 0.36] |  |  |
|  | Unspecified | -0.35 [-1.38, 0.69] |  |  |
| Sex |  |  | F(3, 2.04) = 0.34 | P=0.80 |
|  | Male | -0.23 [-0.74, 0.28] |  |  |
|  | Female | -0.21 [-0.95, 0.54] |  |  |
|  | Mixed/Unspecified | -1.88 [-25.30, 21.54] |  |  |
| Test duration |  |  | F(2, 4.13) = 2.06 | P=0.24 |
|  | Short | -0.55 [-1.18, 0.07] |  |  |
|  | Long | 0.27 [-0.72, 1.27] |  |  |
| Training status |  |  | F(2, 6.08) = 1.22 | P=0.36 |
|  | Active | 0.02 [-1.00, 1.03] |  |  |
|  | Trained | -0.52 [-1.20, 0.16] |  |  |
| Age |  | -0.08 [-0.44, 0.28] | F(1, 6.15) = 0.29 | P=0.61 |

**Supplementary table 5.** Moderators analysis for relative peak power

| **Moderator** | **group** | **SMD [95%CI]** | **F (df1, df2)** | **p** |
| --- | --- | --- | --- | --- |
| Time of day |  |  | F(3, 2.4) = 3.19 | P=0.22 |
|  | Morning | 0.83 [-0.58, 2.23] |  |  |
|  | Evening | 0.78 [0.008, 1.56] |  |  |
|  | Unspecified | 0.35 [-0.008, 0.70] |  |  |
| Training status |  |  | F(2, 4.61) = 9.30 | P=0.02 |
|  | Active | 0.63 [-0.41, 1.68] |  |  |
|  | Trained | 0.45 [0.19, 0.72] |  |  |
| Age |  | -0.09 [-0.19, 0.01] | F(1, 2.34) = 10.47 | P=0.07 |

**Supplementary table 6.** Moderators analysis for relative mean power

| **Moderator** | **group** | **SMD [95%CI]** | **F (df1, df2)** | **p** |
| --- | --- | --- | --- | --- |
| Time of day |  |  | F(3, 2.48) = 4.12 | P=0.17 |
|  | Morning | 0.70 [-0.68, 2.08] |  |  |
|  | Evening | 0.59 [0.11, 1.06] |  |  |
|  | Unspecified | 0.25 [0.002, 0.50] |  |  |
| Test duration |  |  | F(2, 1.69) = 4.43 | P=0.21 |
|  | Short | 0.42 [0.16, 0.67] |  |  |
|  | Longue | 0.19 [-3.63, 4.00] |  |  |
| Training status |  |  | F(2, 6.05) = 6.72 | P=0.03 |
|  | Active | 0.46 [-0.07, 0.98] |  |  |
|  | Trained | 0.31 [0.07, 0.56] |  |  |
| Age |  | -0.09 [-0.23, 0.05] | F(1, 2.78) = 4.39 | P=0.13 |
| Exposure |  | 0.04 [-0.30, 0.37] | F(1,1.43)= 0.47 | P=0.59 |

**Supplementary table 7.** Moderators analysis for jump height

| **Moderator** | **group** | **SMD [95%CI]** | **F (df1, df2)** | **p** |
| --- | --- | --- | --- | --- |
| Selection |  |  | F(2, 0.82) = 0.67 | P=0.67 |
|  | SSM | 3.52 [-6.17, 13.21] |  |  |
|  | PSM | 1.84 [-1.64, 5.32] |  |  |
| Tempo |  |  | F(2, 1.54) = 0.75 | P=0.59 |
|  | Fast | 3.25 [-3.51, 10.02] |  |  |
|  | Slow | 0.76 [-2.71, 4.23] |  |  |
| Sex |  |  | F(2, 1) = 3649.66 | P=0.01 |
|  | Male | 3.04 [-3.12, 9.20] |  |  |
|  | Female | 0.10 [0.09, 0.11] |  |  |
| Age |  | -0.89 [-4.70, 2.93] | F(1, 1.58) = 1.71 | P=0.35 |

**Supplementary table 8.** Moderators analysis for perceived exertion

| **Moderator** | **group** | **SMD [95%CI]** | **F (df1, df2)** | **p** |
| --- | --- | --- | --- | --- |
| Selection |  |  | F(3, 2.56) = 2.92 | P=0.22 |
|  | SSM | -0.49 [-0.92, -0.07] |  |  |
|  | PSM | 0.22 [-0.03, 0.48] |  |  |
|  | Pseudo | -0.25 [-2.20, 1.69] |  |  |
| Tempo |  |  | F(2, 3.56) = 1.53 | P=0.33 |
|  | Fast | -0.37 [-0.95, 0.20] |  |  |
|  | Slow | -0.52 [-1.70, 0.65] |  |  |
| Time of day |  |  | F(3, 7.08) = 0.05 | P=0.99 |
|  | Morning | 0.12 [-1.06, 1.30] |  |  |
|  | Evening | -0.01 [-0.36, 0.34] |  |  |
|  | Unspecified | -0.02 [-0.38, 0.34] |  |  |
| Timing of measure |  |  | F(3, 2.66) = 6.38 | P=0.10 |
|  | During | -0.41 [-1.35, 0.52] |  |  |
|  | Post-warm up | -0.43 [-1.20, 0.34] |  |  |
|  | After | 0.20 [-0.28, 0.69] |  |  |
| Sex |  |  | F(3, 3.62) = 2.32 | P=0.23 |
|  | Male | 0.11 [-0.22, 0.43] |  |  |
|  | Female | -0.03 [-0.41, 0.35] |  |  |
|  | Mixed/Unspecified | -0.44 [-0.98, 0.10] |  |  |
| Test duration |  |  | F(2, 8.43) = 0.18 | P=0.84 |
|  | Short | 0.04 [-0.28, 0.37] |  |  |
|  | Longue | -0.11 [-0.68, 0.46] |  |  |
| Training status |  |  | F(2, 8.8) = 2.44 | P=0.14 |
|  | Active | -0.38 [-1.09, 0.33] |  |  |
|  | Trained | 0.20 [-0.04, 0.43] |  |  |
| Age |  | -0.07 [-0.15, 0.005] | F(1, 6.17) = 5.17 | P=0.06 |
| Exposure duration |  | 0.07 [-0.15, 0.29] | F(1, 1.23) = 6.63 | P=0.20 |

**Supplementary table 9.** Moderators analysis for feeling scale

| **Moderator** | **group** | **SMD [95%CI]** | **F (df1, df2)** | **p** |
| --- | --- | --- | --- | --- |
| Selection |  |  | F(2, 1.78) = 66.57 | P=0.02 |
|  | SSM | 2.68 [-5.78, 11.14] |  |  |
|  | PSM | 2.35 [1.36, 3.34] |  |  |
| Tempo |  |  | F(2, 1.45) = 7.12 | P=0.18 |
|  | Fast | 2.72 [-0.05, 5.49] |  |  |
|  | Slow | 1.78 [-0.33, 3.89] |  |  |
| Timing of measure |  |  | F(2, 1.79) = 32.57 | P=0.04 |
|  | Post-warm up | 4.65 [-1.80, 11.10] |  |  |
|  | After | -0.26 [-6.37, 5.86] |  |  |
| Test duration |  |  | F(2, 1.10) = 2.57 | P=0.38 |
|  | Short | 2.74 [-3.27, 8.76] |  |  |
|  | Longue | 1.96 [-6.76, 10.68] |  |  |
| Training status |  |  | F(2, 1.03) = 596.13 | P=0.03 |
|  | active | 0.15 [0.11, 0.19] |  |  |
|  | trained | 2.77 [0.12, 5.41] |  |  |
| Time of day |  |  | F(2, 1.10) = 5.35 | P=0.39 |
|  | Morning | 3.80 [-1.15, 8.76] |  |  |
|  | Evening | 2 [-5.19, 9.18] |  |  |
|  | Unspecified | 0.13 [0.03, 0.23] |  |  |
| Age |  | -0.33 [-2.32, 1.65] | F(1, 1.15) = 2.46 | P=0.34 |

**Supplementary table 10.** Moderators analysis for fatigue symptoms

| **Moderator** | **group** | **SMD [95%CI]** | **F (df1, df2)** | **p** |
| --- | --- | --- | --- | --- |
| Selection |  |  | F(2, 0.88) = 23.23 | P=0.17 |
|  | SSM | -0.17 [-0.75, 0.41] |  |  |
|  | PSM | -0.24 [-0.40, -0.09] |  |  |
| Tempo |  |  | F(2, 0.5) = 69.15 | P=0.24 |
|  | Fast | -0.27 [-0.45, -0.09] |  |  |
|  | Slow | -0.17 [-0.53, 0.20] |  |  |
| Training status |  |  | F(2, 0.5) = 8.93 | P=0.41 |
|  | active | -0.22 [-0.64, 0.19] |  |  |
|  | trained | -0.20 [-1.13, 0.74] |  |  |
| Age |  | 0.02 [-0.01, 0.05] | F(1, 1.7) = 6.79 | P=0.14 |

**Supplementary table 11.** Results of the fitted model using the 0.5 and 0.9 correlation coefficients

| **Correlation/**  **Outcome** | **R= 0.5** | | | | | **R= 0.9** | | | | |
| --- | --- | --- | --- | --- | --- | --- | --- | --- | --- | --- |
|  | **SMD [95% CI]** | **p** | **Q (df)** | **I² (%)** | **PI** | **SMD [95% CI]** | **p** | **Q (df)** | **I² (%)** | **PI** |
| RPE | 0.005[-0.26; 0.28] | 0.97 | Q(104)= 446.55 | 83.43% | [-1.55; 1.56] | 0.007[-0.26; 0.28] | 0.96 | Q(104)= 912.11 | 95.54% | [-1.63; 1.1.64] |
| RMP | 0.38[0.15;0.60] | 0.004 | Q(20)= 39.76 | 41.28% | [-0.24; 0.99] | 0.39 [0.16;0.61] | 0.003 | Q(20)= 106.89 | 84.72% | [-0.47; 1.24] |
| RPP | 0.54[0.21; 0.87] | 0.005 | Q(17)= 41.18 | 59.92% | [-0.41; 1.49] | 0.53 [0.21; 0.85] | 0.004 | Q(17) = 97.49 | 86.66% | [-0.50; 1.56] |
| PDI | -0.39[-0.92; 0.14] | 0.14 | Q(49) = 344.20 | 92.28% | [-2.88; 2.10] | -0.38[-0.91; 0.14] | 0.14 | Q(49) = 593.72 | 97.84% | [-2.91; 2.15] |
| Jump height | 2.25 [-1.74; 6.23] | 0.17 | Q(15) = 186.13 | 98.09 % | [-7.03, 11.52] | 2.24 [-1.74; 6.22] | 0.17 | Q(15) = 225.93 | 99.55% | [-7.05; 11.53] |
| FS | 2.42 [0.52; 4.31] | 0.03 | Q(23) = 406.11 | 99.11% | [-11.41; 16.25] | 2.42 [0.52; 4.31] | 0.03 | Q(23) = 494.88 | 99.73% | [-11.44; 16.28] |
| Completion time | -0.27 [-0.52;-0.02] | 0.04 | Q(30) = 96.36 | 38 % | [-0.89; 0.36] | -0.24 [-0.47;-0.01] | 0.04 | Q(30) = 159.55 | 79.48% | [-0.97; 0.49] |
| Fatigue symptoms | -0.21[-0.33; -0.09] | 0.01 | Q(7) = 5.3247 | 9.17% | [-0.33; -0.09] | -0.21 [-0.32; -0.10] | 0.008 | Q(7) = 19.74 | 6.7 % | [-0.81; 0.39] |

FS: feeling scale; RPE: rate of perceived exertion; PDI: performance decrement index; RPP: relative peak power; RMP: relative mean power; PI: prediction interval; ES: effect size.
